# Shiga toxin remodels the intestinal epithelial transcriptional response to Enterohemorrhagic *Escherichia coli*

**DOI:** 10.1371/journal.ppat.1009290

**Published:** 2021-02-02

**Authors:** Alyson R. Warr, Carole J. Kuehl, Matthew K. Waldor

**Affiliations:** 1 Division of Infectious Diseases, Brigham & Women’s Hospital, Boston, Massachusetts, United States of America; 2 Department of Microbiology, Harvard Medical School, Boston, Massachusetts, United States of America; 3 Howard Hughes Medical Institute, Boston, Massachusetts, United States of America; University of Pennsylvania, UNITED STATES

## Abstract

Enterohemorrhagic *Escherichia coli* (EHEC) is a food-borne pathogen that causes diarrheal disease and the potentially lethal hemolytic uremic syndrome. We used an infant rabbit model of EHEC infection that recapitulates many aspects of human intestinal disease to comprehensively assess colonic transcriptional responses to this pathogen. Cellular compartment-specific RNA-sequencing of intestinal tissue from animals infected with EHEC strains containing or lacking Shiga toxins (Stx) revealed that EHEC infection elicits a robust response that is dramatically shaped by Stx, particularly in epithelial cells. Many of the differences in the transcriptional responses elicited by these strains were in genes involved in immune signaling pathways, such as *IL23A*, and coagulation, including *F3*, the gene encoding Tissue Factor. RNA FISH confirmed that these elevated transcripts were found almost exclusively in epithelial cells. Collectively, these findings suggest that Stx potently remodels the host innate immune response to EHEC.

## Author summary

Enterohemorrhagic *Escherichia coli* (EHEC) is a potentially lethal foodborne pathogen. During infection, EHEC releases a potent toxin, Shiga toxin (Stx), into the intestine, but there is limited knowledge of how this toxin shapes the host response to infection. We used an infant rabbit model of infection that closely mimics human disease to profile intestinal transcriptomic responses to EHEC infection. Comparisons of the transcriptional responses to infection by strains containing or lacking Stx revealed that this toxin markedly remodels how the epithelial cell compartment responds to infection. Our findings suggest that Stx shapes the intestinal innate immune response to EHEC and provide insight into the complex host-pathogen dialogue that underlies disease.

## Introduction

Enterohemorrhagic *Escherichia coli* (EHEC) is an important foodborne pathogen responsible for up to 2 million annual cases of acute gastrointestinal illness [1]. Chiefly colonizing the colon, EHEC typically leads to self-limited hemorrhagic colitis; however, 5–10% of infected individuals also develop hemolytic uremic syndrome (HUS), a potentially life-threatening complication that can lead to renal failure [2,3]. Supportive rehydration therapy remains the primary treatment for EHEC infection, as antibiotics are associated with elevated frequencies of HUS and therefore contraindicated [4]. There is no vaccine available. EHEC infection is associated with an inflammatory response in the colon, and patients have elevated fecal leukocytes and calprotectin levels. Colonic biopsy samples from patients with EHEC infection exhibit inflammation, edema, fibrin deposition, neutrophil invasion, and hemorrhage [5–9].

*E*. *coli* O157:H7 is the most common EHEC serotype, but other serotypes have been described. All serotypes share two primary virulence factors: the ‘LEE’ pathogenicity island that encodes a type III secretion system (T3SS), and prophages that encode one or more Shiga toxins [10]. The activities of EHEC’s T3SS effector proteins mediate the pathogen’s tight adherence to the colonic mucosa [11], and can promote or antagonize the inflammatory response in epithelial cells [12–15].

During infection, EHEC produces and releases Shiga toxins (Stx) into the intestinal lumen. Stxs are potent AB_5_ subunit exotoxins which can bind to the host cell surface glycosphingolipid globotriaosylceramide (Gb3). Once internalized into the eukaryotic cell cytosol, Stx catalyzes a site-specific depurination of the 28s rRNA, which leads to inhibition of protein synthesis and triggers the ribotoxic stress response, production of cytokines, and cell death [16–20]. Absorption of Stx into the blood results in its systemic circulation and damage to endothelial cells, particularly in the renal microvasculature, leading to the characteristic findings of HUS.

The role of Stx in EHEC pathogenicity in the colon is controversial. Although Stx has been associated with colonic pathology [5,6,21–23], the mechanisms that explain these observations are unclear because the presence of Gb3 in the colonic epithelium has been disputed [24–26,22]. Some suggest that Stx does not directly act on the colonic epithelium, but instead passes through the epithelial layer to primarily act on endothelial and immune cells [27–31]. Stx may also enter colon epithelial cells through binding alternative receptors [26,32]. Despite the ambiguity surrounding the mechanisms by which Stx exerts toxic effects in the colon during EHEC infection, purified Stx can stimulate inflammatory responses in cultured cells [33–36,17,22,37–40,20,19]. Analyses of the transcriptional responses to EHEC infection in cultured epithelial cells and organoids have also demonstrated that processes linked to inflammatory signaling, cytoskeletal organization, and apoptosis are altered [41–44]. However, to date, our knowledge of the host response to EHEC infection is almost exclusively derived from tissue-culture based studies. Because mice do not develop overt diarrhea or colonic pathology during EHEC infection [45], no comprehensive in vivo analyses of how EHEC modifies colonic gene expression patterns during infection have been reported. Moreover, the extent to which Stx contributes to such gene expression changes in vivo is unclear.

Here, we used infant rabbits, a small animal model of EHEC infection where orogastric inoculation of the pathogen leads to a disease that closely mimics the intestinal manifestations of human EHEC disease [21,23,46], to investigate how Stx production in the gut modifies the cellular response of the colonic mucosa during EHEC infection. We compared the colonic epithelial and lamina propria transcriptional responses to WT and mutant EHEC lacking Stx genes. Collectively, our findings provide a comprehensive profile of the colonic transcriptional responses to EHEC and suggest that Stx markedly remodels the gene expression of epithelial cells.

## Results

### Shiga toxin promotes apoptosis and hemorrhage in the colonic mucosa

We used EDL933, a prototypical *E*. *coli* O157:H7 clinical isolate (WT) [47,48], to investigate how Shiga toxins modify the response of the colonic mucosa to EHEC infection. This strain encodes two Stx variants, Stx1 and Stx2, which were both deleted to yield strain ΔΔ*stx*. WT and ΔΔ*stx* were orogastrically administered to infant rabbits to determine if Stx influences EHEC intestinal colonization or the colonic mucosal response to infection. As described previously in experiments with a different EHEC clinical isolate [21], the burden of WT and ΔΔ*stx* in the colon did not differ (S1 Fig), suggesting that Stx does not alter EHEC colonization in this model. Nonetheless, Stx appeared to contribute to the development of diarrhea as described (89% diarrhea in WT-inoculated animals vs 40% in ΔΔ*stx-*inoculated animals) [21].

**Fig 1:**
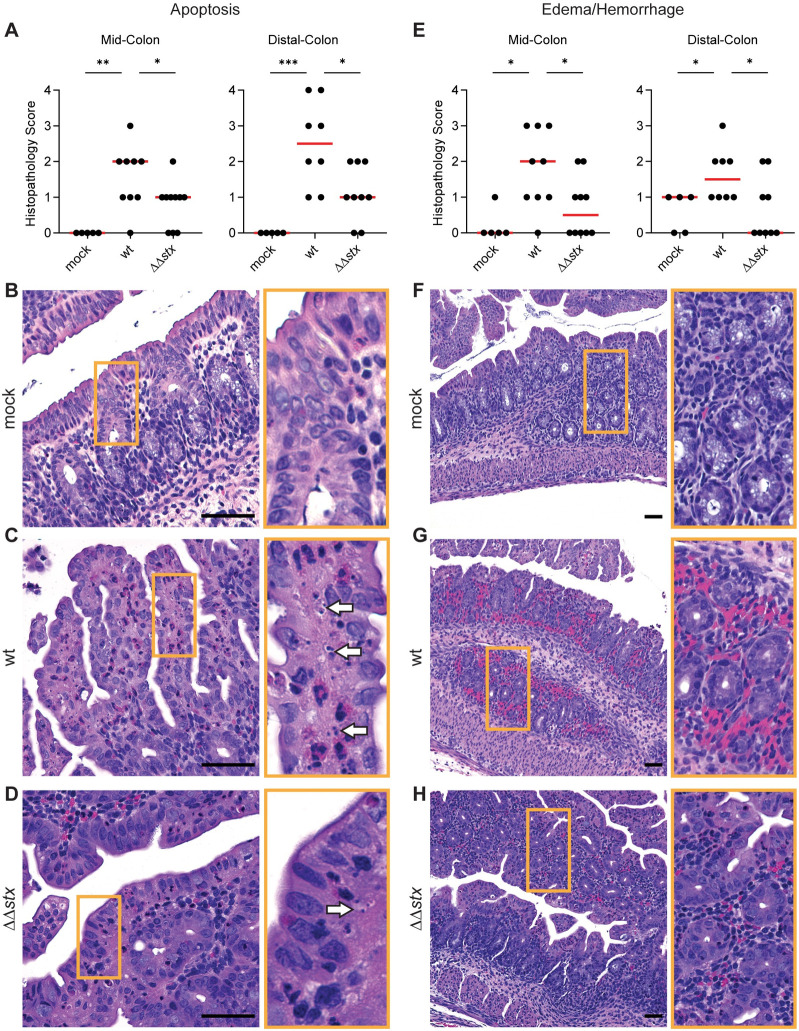
Apoptosis and hemorrhage/edema in the colon is more prominent in animals infected with WT vs ΔΔ*stx* EHEC. (A) Apoptotic nuclei in colon sections from infant rabbits inoculated with PBS (mock), WT or ΔΔ*stx* EHEC. Scores for individual animals are plotted with the median (red line). Statistical comparisons were made using a Mann-Whitney U, p<0.05 (*), 0.01 (**), or 0.001 (***). (B-D): Example images from mock (score = 0), WT (score = 4), and ΔΔ*stx* (score = 2) infected colons. White arrows indicate apoptotic nuclei. (E): Severity of hemorrhage/edema. (F-H): Representative example images from mock (score = 0), WT (score = 3) and ΔΔ*stx* (score = 1) infected colons. Scale bars indicate 50 μm. Orange boxes denotes inset.

Histopathologic analyses of colon samples from animals inoculated with WT, ΔΔ*stx*, or PBS (mock) were carried out at peak colonization (36–40 hours post inoculation). Compared to mock-treated control animals, colon samples from both WT and ΔΔ*stx* infected rabbits had prominent pathologic changes in the mid and distal colon, sites of maximal colonization. WT and ΔΔ*stx* infections led to similar levels of overall colonic inflammation, characterized by an increased number of small mononuclear cells in the submucosal tissue, as well as comparable levels of heterophil (lapine neutrophil) infiltration (S2 Fig). Both WT and ΔΔ*stx* infection elicited minor epithelial sloughing in the colon (S3 Fig). However, compared to the colonic pathology associated with the ΔΔ*stx* strain, there was significantly more apoptosis, indicated by widespread fragmented nuclei, and edema/hemorrhage, indicated by widespread blood and fluid accumulation in tissue, observed in samples from animals infected with WT EHEC (Fig 1). These observations are consistent with previous descriptions of histopathologic changes associated with other EHEC strains in this model [21] and also support the hypothesis that EHEC production of Stx in the intestine provokes local pathology including apoptosis and hemorrhage in the colonic mucosa.

### Shiga toxin shapes the colonic mucosal transcriptomic response to EHEC

To further investigate how Stx modifies the colonic mucosa’s response to EHEC infection, we used RNA-seq to characterize the transcriptomes of colonic epithelial and lamina propria cells derived from infant rabbits orogastrically inoculated with WT, ΔΔ*stx*, or PBS (mock). In the colon, epithelial cells make initial contact with EHEC and Stx. Beneath the epithelial layer, cells in the lamina propria, including stromal and immune cells, respond to signals from the epithelial cells or potentially from direct contact with PAMPs to trigger additional immune responses [49]. To profile transcriptional changes in these cell populations, we harvested colons at the time of peak colonization and performed enrichment protocols for epithelial and lamina propria cells from 3 rabbits per inoculum type.

RNA was extracted from these cell fractions, subjected to next-generation sequencing, and mapped to the rabbit genome. Normalized expression of marker genes associated with epithelial, stromal, and immune cells were compared between epithelial and lamina propria fractions (S4 Fig). Epithelial cell markers, such as *EPCAM*, *VIL1*, and *MUC1* were enriched in the epithelial cell fraction (S4A Fig), and stromal cell markers such as *COL1A* and immune cell markers such as *PTPRC* were enriched in the lamina propria fraction (S4B and S4C Fig), confirming the enrichment of desired cell populations. We were also able to detect enrichment in the Gb3-synthase *A4GALT* [50] in the epithelial cell fraction (S4D Fig), validating previous data that the Stx receptor is present in infant rabbit tissue [23].

**Fig 2:**
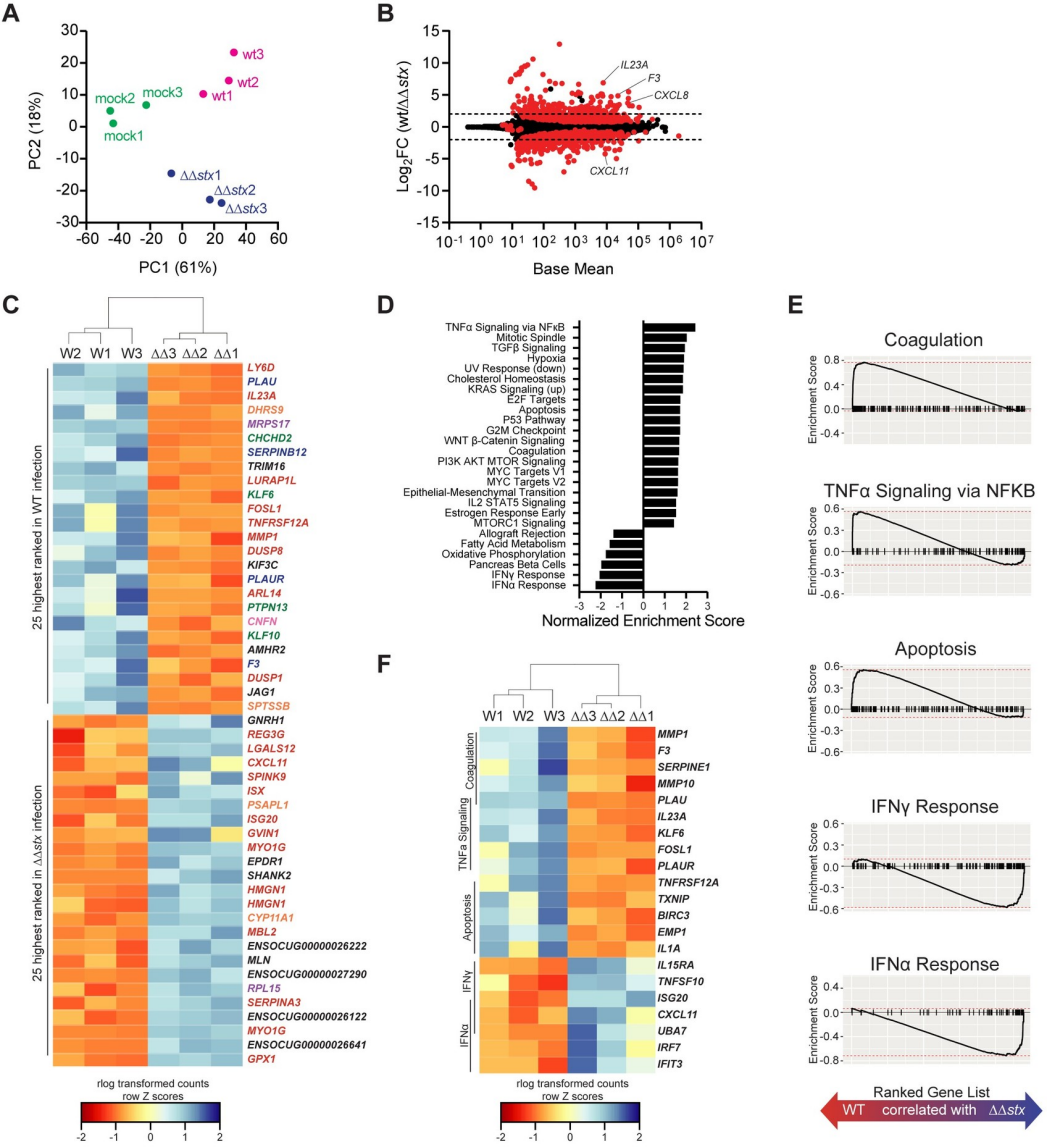
Profiles of colonic epithelial cell transcriptional responses differ between animals infected with WT and ΔΔ*stx* EHEC. (A) Principal component analysis of rlog-transformed expression values. (B) Average expression level (base mean) and log2 fold change of transcript abundance from rabbits inoculated with WT or ΔΔ*stx* EHEC. Red dots are genes with significantly different (p<0.05) transcript abundance. Dashed line indicates log2 fold change >2 or <-2. (C) Heat map of rlog-transformed read counts for top 25 and bottom 25 genes by rank. Rows are Z-normalized. Gene names are colored by function: red–immune, orange–metabolism, green–proliferation/apoptosis, blue–coagulation, purple–translation/protein folding, pink–barrier function/cytoskeleton, black–uncharacterized or other. (D) Hallmark gene sets significantly associated with WT or ΔΔ*stx* infection. (E) Gene set enrichment plot for selected pathways. Black tick marks are genes within pathway organized by rank. (F) Heat map of rlog-transformed read counts for top 5 genes in the leading edge for indicated pathway organized by rank.

**Fig 3:**
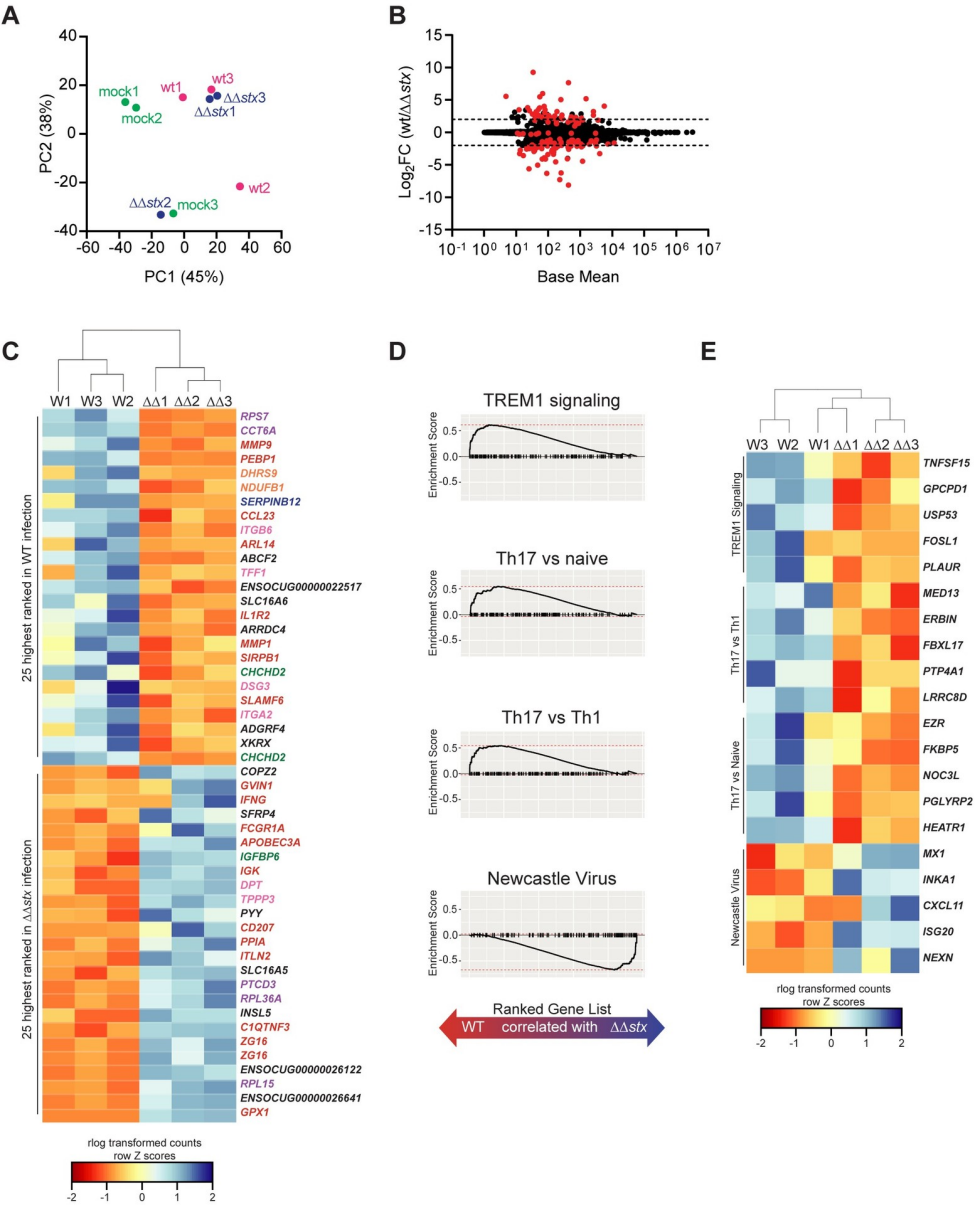
Profiles of colonic lamina propria cell transcriptional responses differ between animals infected with WT and ΔΔ*stx* EHEC. (A) Principal component analysis of rlog-transformed expression values of lamina propria cells. (B) Average expression level (base mean) and log2 fold change of transcript abundance from rabbits inoculated with WT or ΔΔ*stx* EHEC. Red dots are genes with significantly different (p<0.05) transcript abundance. Dashed line indicates log2 fold change >2 or <-2. (C) Heat map of rlog-transformed read counts for top 25 and bottom 25 genes by rank. Rows are Z-normalized. Gene names are colored by function: red–immune, orange–metabolism, green–proliferation/apoptosis, blue–coagulation, purple–translation/protein folding, pink–barrier function/cytoskeleton, black–uncharacterized or other. (D) Gene set enrichment plot for selected pathways. Black tick marks are genes within pathway organized by rank. (E) Heat map of rlog-transformed read counts for top 5 genes in the leading edge for indicated pathway organized by rank.

Next, the global gene expression profiles for individual samples were evaluated and compared using the differential gene expression package DESeq2. Principal component analysis (PCA) revealed that epithelial samples from each group clustered separately, highlighting the specificity of the transcriptomic responses elicited by each inoculation type in this compartment (Fig 2A). PCA did not segregate the lamina propria samples as neatly based on the presence or absence of *stx* (Fig 3A), suggesting that the transcriptional response to Stx is concentrated in the epithelial compartment.

Relative to the mock infected samples, WT infection stimulated more transcriptomic changes than ΔΔ*stx* infection in colonic epithelial cells. Of the ~30,000 rabbit genes surveyed, 1126 had significantly different expression in WT/mock; for ΔΔ*stx*/mock, 497 genes were differentially expressed (S5 Fig and S1 and S2 Tables). 321 genes were similarly differentially expressed in both infection types (S6A Fig). Most of these shared genes were associated with GO Molecular Function terms commonly linked to bacterial infection, including cytokine/chemokine signaling and binding, and LPS recognition through TLR4 signaling (S6B Fig), implying that many typical pathogen-response pathways are activated similarly in the presence or absence of Stx. Similar patterns were observed in the lamina propria transcriptome (S6C and S6D Fig and S3 and S4 Tables).

Although the WT and ΔΔ*stx* strains stimulated a set of shared transcripts, we also found robust and widespread differences in the epithelial transcriptomic responses to WT vs ΔΔ*stx* infection. 390 genes exhibited significantly different expression between these two conditions (Fig 2B and S5 Table). Genes were ranked by adjusted p-value, and hierarchical clustering was performed on rlog transformed read counts. The clustering analysis confirmed that WT and ΔΔ*stx* infection elicit markedly distinct transcriptomic signatures in the epithelium (S7A Fig). Many of the top 50 genes by rank were involved in processes related to coagulation and immune signaling (Fig 2C). Gene set enrichment analysis (GSEA) was performed to further identify transcriptional processes associated with WT or ΔΔ*stx* infection. We identified a number of pathways specifically associated with WT or ΔΔ*stx* infection (Fig 2D and S5 Table). Notably, apoptosis, coagulation, and NFκB signaling were associated with the transcriptomic response to WT infection, whereas pathways linked with IFNα and IFNγ signaling were associated with ΔΔ*stx* infection (Fig 2E). Using hierarchical clustering on rlog transformed counts of the top five genes from each enrichment-driving leading-edge subset, we observed dramatic differences between the transcriptional responses to WT and ΔΔ*stx* infection (Fig 2F). Specifically, drivers of coagulation, including the gene coding for tissue factor *F3*, as well as pro-inflammatory cytokines *IL23A* and *IL1A* were specifically associated with WT infection (Fig 2F). In ΔΔ*stx* infection, many interferon-stimulated genes (ISGs) were differentially upregulated, including the T-cell chemokine *CXCL11*. Collectively, these analyses suggest that Stx shapes the epithelial cell innate immune response to EHEC.

In samples from the lamina propria, far fewer genes exhibited differential expression in comparisons between WT and ΔΔ*stx*-infections than in epithelial samples (91 vs 390) (Fig 3B and S6 Table). Similar to the PCA analysis (Fig 3A), hierarchical clustering of the full transcriptome did not separate the transcriptional profiles of WT and ΔΔ*stx*-infected colons as clearly as observed in epithelial samples (S7A and S7B Fig). However, clustering analysis showed that the top 50 genes by rank were distinguishable by inoculum type (Fig 3C). Many of these genes function in coagulation and immune signaling pathways, including several matrix metalloproteases (MMPS), IFNγ, ISGs, and *SLAMF6* and *CD207* (Fig 3C). GSEA was performed using gene sets defined by the Immune Signatures Database (S6 Table) [51]. Pathways of interest associated with WT infection included “Th17 vs Th1,” “Th17 vs Naive,” and “TREM1 signaling” and revealed that signatures associated with immune responses to extracellular pathogens are present in the lamina propria (Fig 3D). Gene-sets associated with ΔΔ*stx* infection included “Newcastle Virus,” which induces a strong interferon response (Fig 3D). Examining the leading-edge subset of these pathways underscored that genes associated with Th17 cells are associated with WT infection while IFNγ-stimulated genes such as *CXCL11* are associated with ΔΔ*stx* infection in the lamina propria (Fig 3E). Together, these analyses suggest that in the lamina propria, Stx induces the expression of genes typically associated with type 3 immune cells and that in the absence of this toxin, IFN-related pathways are more prominent.

### EHEC stimulates expression of coagulation-associated genes in a Stx-dependent manner

Comparison of expression profiles from epithelial and lamina propria samples from animals infected with WT vs ΔΔ*stx* EHEC revealed differences in many genes associated with coagulation. Samples from WT infection had higher levels of transcripts for *F3*, a gene encoding the initiator of the clotting cascade, MMPs and SERPINs, which are proteases that regulate the processing of coagulation cascade proteins, as well as urokinase and the urokinase receptor, which regulate fibrin deposition. Stx is known to cause thrombosis in vascular beds outside of the GI tract and has been investigated in the kidney microvasculature [20,52,53], but comparatively few studies have focused on Stx-linked coagulation in the intestine.

**Fig 4:**
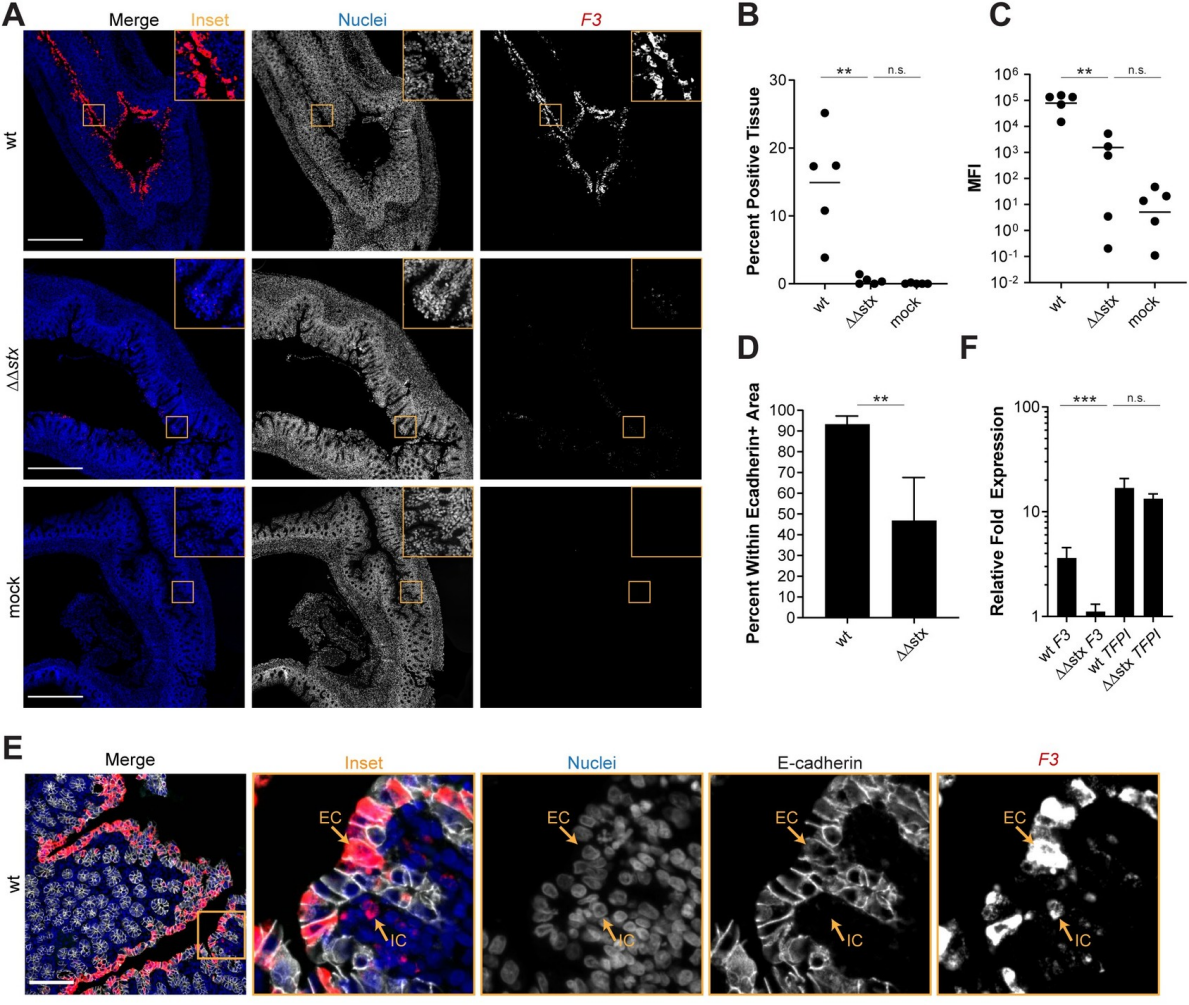
Expression of *F3* in epithelial cells is much greater in animals infected with WT vs ΔΔ*stx* EHEC. (A) Micrographs of colon sections from rabbits inoculated with WT EHEC, ΔΔ*stx* EHEC, or PBS (mock) stained with a probe to rabbit *F3* mRNA (red) and DAPI (blue). Scale bar is 500 μM. (B) Percentage of tissue section with *F3* signal from individual colons. Distributions compared using Mann-Whitney U test, p<0.01 (**), n.s. indicates not significant. (C) Mean fluorescent intensity (MFI) from individual colons plotted with mean. Distributions compared using Mann-Whitney U test, p<0.01 (**), n.s. indicates not significant. (D) Percent *F3* signal within E-cadherin positive cells. Distributions compared using the Mann-Whitney U test, p<0.05 (*). (E) Sections stained with a probe to rabbit *F3* mRNA (red), DAPI (blue), and anti-E-cadherin antibody (white). Scale bar is 500 μM. Example immune cell (IC) and epithelial cell (EC) are indicated. (F) Normalized expression of *F3* and *TFPI* in HT29 cells infected with WT EHEC, ΔΔ*stx* EHEC, or PBS. Expression levels compared with a Students two-tailed t-test, p<0.001 (***), n.s. indicates not significant.

As antibodies to detect rabbit proteins in tissue are not readily available, we used RNA FISH to investigate the localization of transcripts of interest identified in the RNAseq data. We probed for Tissue Factor (*F3*) transcripts in infected and control samples and found that there was markedly greater *F3* expression in samples from WT-infected vs ΔΔ*stx*-infected or control rabbits (Fig 4A). *F3* transcripts were observed in >10% of total DAPI+ colon tissue in WT infection and in only ~0.1% of tissue in ΔΔ*stx* infected animals (Fig 4B). Mean fluorescence intensity (MFI), a proxy for transcript abundance, was much higher in WT vs ΔΔ*stx* samples and the MFI in the latter samples did not differ from the values found in mock infected samples (Fig 4C), suggesting that Stx is critical for stimulating *F3* expression. Notably, almost all (>90%) of the *F3*-hybridizing signal in WT samples was detected in cells expressing E-Cadherin, an epithelial marker (Fig 4D and 4E). The uniformity of *F3* expression along the epithelium, even in sections where few EHEC cells were detected (S8 Fig), suggests that Stx may diffuse along the epithelium to modify transcriptional programs in cells that do not have attached EHEC; alternatively, epithelial cells with attached EHEC may secrete factors that modify transcription in neighboring cells.

We also explored whether Stx stimulates *F3* transcription in HT29 cells, a human colonic epithelial cell line. qPCR was used to quantify *F3* transcripts in HT29 cells after infection with WT, ΔΔ*stx* EHEC, or PBS (mock). With WT infection, *F3* gene expression was 5-fold higher than in uninfected cells (Fig 4F); moreover, as in rabbits, induction of *F3* expression in HT29 cells was largely dependent on Stx and there was little difference in *F3* expression in uninfected cells vs those infected with ΔΔ*stx* infection (Fig 4F). However, the ability of the ΔΔ*stx* mutant to induce *F3* in HT29 cells was restored by addition of pure Stx2 (S9A Fig). Stx2 itself, without bacteria, also induced *F3* expression (S9A Fig). It has been reported that Stx leads to increases in Tissue Factor pro-coagulant protein activity in renal proximal tubule cells and endothelial cells [54–57]; this effect is thought to be primarily driven by a decrease in the expression of Tissue Factor Protein Inhibitor (TFPI), and not an increase in *F3* gene expression [54,58]. We also measured *TFPI* transcript levels in the samples used to measure *F3* expression, to assess if this pathway is also active in HT29 cells. Both WT and ΔΔ*stx* infection similarly stimulated expression of *TFPI* transcripts >10-fold compared to the uninfected cells (Fig 4F). Thus, Stx appears to regulate Tissue Factor by different mechanisms in colonic epithelial cells vs endothelial cells.

### Stx alters cytokine gene expression

**Fig 5:**
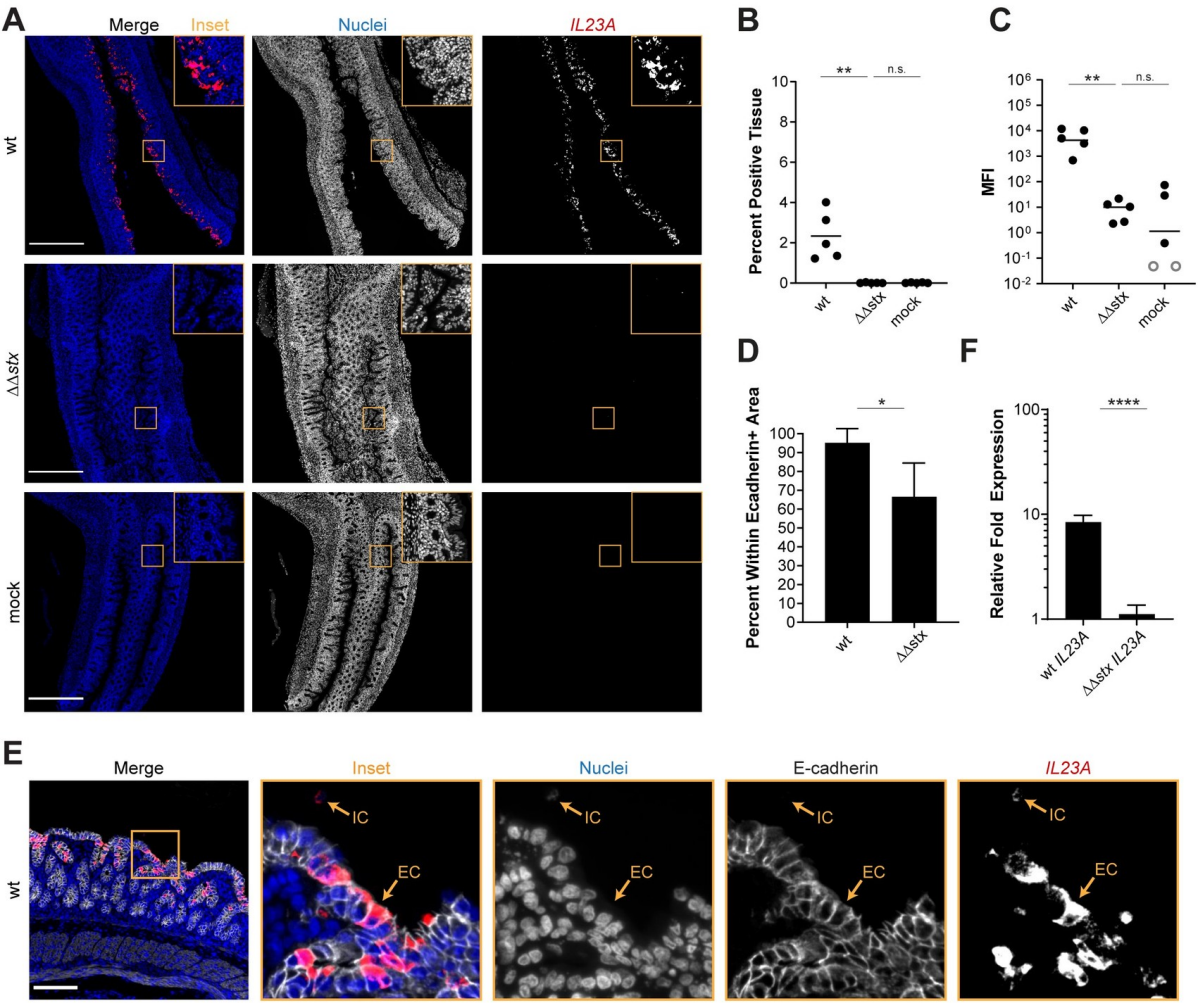
Expression of *IL23A* in epithelial cells is much greater in animals infected with WT vs ΔΔ*stx* EHEC. (A) Micrographs of colon sections from rabbits inoculated with WT EHEC, ΔΔ*stx* EHEC, or PBS (mock) stained with a probe to rabbit *IL23A* mRNA (red) and DAPI (blue). Scale bar is 500 μM. (B) Percentage of tissue section with *IL23A* signal from individual colons. Distributions compared using Mann-Whitney U test, p<0.01 (**), n.s. indicates not significant. (C) Mean fluorescent intensity (MFI) from individual colons plotted with mean. Distributions compared using Mann-Whitney U test, p<0.01 (**), n.s. indicates not significant. (D) Percent *IL23A* signal within E-cadherin positive cells. Distributions compared using the Mann-Whitney U test, p<0.05 (*). (E) Sections stained with a probe to rabbit *IL23A* mRNA (red), DAPI (blue), and anti-E-cadherin antibody (white). Scale bar is 500 μM. Example immune cell (IC) and epithelial cell (EC) is indicated. (F) Normalized expression of *IL23A* in HT29 cells infected with WT EHEC, ΔΔ*stx* EHEC, or PBS. Expression levels compared with a Students two-tailed t-test, p<0.001 (***), n.s. indicates not significant.

Analyses presented above revealed that Stx markedly alters colonic mucosal gene expression during EHEC infection, stimulating expression of several transcripts coding for pro-inflammatory cytokines, such as IL23, relative to levels observed in ΔΔ*stx* infection (Figs 2 and 3). We used RNA FISH to compare the fluorescence intensity and distribution of *IL23A* transcripts in colons from rabbits infected with WT or ΔΔ*stx* (Fig 5A). The intensity and percent of tissue expressing *IL23A* signal in samples from animals infected with WT EHEC were strikingly higher than those in animals infected with ΔΔ*stx* EHEC (Fig 5B and 5C). These values did not differ in ΔΔ*stx* and control samples, suggesting that Stx stimulates *IL23A* expression. Unexpectedly, nearly all of the *IL23A* expression in WT samples was detected within epithelial cells (E-cadherin positive cells) compared to cells in the lamina propria (Fig 5D). We validated this finding using tissue-cultured HT29 cells; *IL23A* expression was induced in cells infected by WT but not ΔΔ*stx* (Fig 5F). Similar to the findings above with *F3* expression, addition of pure Stx2 restored ΔΔ*stx*’s capacity to stimulate *IL23A* expression to WT levels (S9B Fig). Stx2 by itself also induces *IL23A* (S9B Fig).

IL23 also promotes the expression of other cytokines such as the chemokine CXCL8, which is a neutrophil chemoattractant. Similar to *F3* and *IL23A* expression, *CXCL8* transcripts were observed at much greater intensity and in a much larger area of tissue in samples from WT vs ΔΔ*stx* infection (S8A–S8C Fig). Furthermore, these *CXCL8* transcripts were primarily present in epithelial cells (S10D–S10F Fig). Also similar to *F3*, *CXCL8* and *IL23A* signals were uniform throughout epithelial tissue, showing no apparent correlation with EHEC foci along the epithelium (S10 Fig). Together these observations suggest that Stx stimulates expression of cytokine-related genes in the colonic epithelium during EHEC infection.

**Fig 6:**
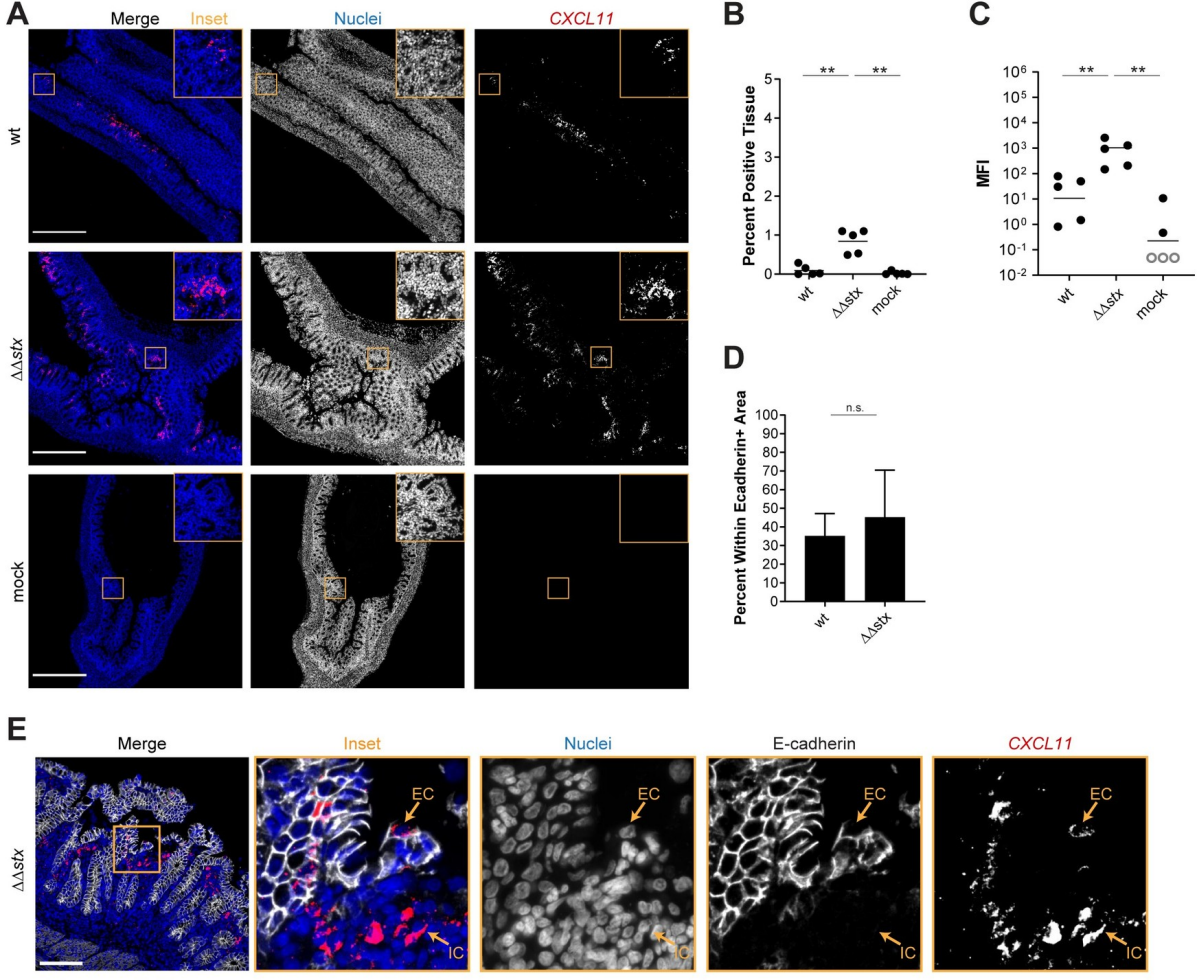
Expression of *CXCL11* in is greater in animals infected with ΔΔ*stx* vs WT EHEC. (A) Micrographs of colon sections from rabbits inoculated with WT EHEC, ΔΔ*stx* EHEC, or PBS (mock) stained with a probe to rabbit *CXCL11* mRNA (red) and DAPI (blue). Scale bar is 500 μM. (B) Percentage of tissue section with *CXCL11* signal from individual colons. Distributions compared using Mann-Whitney U test, p<0.01 (**), n.s. indicates not significant. (C) Mean fluorescent intensity (MFI) from individual colons plotted with mean. Distributions compared using Mann-Whitney U test, p<0.01 (**), n.s. indicates not significant. (D) Percent *CXCL11* signal within E-cadherin positive cells. Distributions compared using the Mann-Whitney U test, p<0.05 (*). (E) Sections stained with a probe to rabbit *CXCL11* mRNA (red), DAPI (blue), and anti-E-cadherin antibody (white). Scale bar is 500 μM. Example immune cell (IC) and epithelial cell (EC) is indicated.

In the RNAseq data, we found that animals infected with ΔΔ*stx* had statistically significant higher levels of *IFNG* transcripts than those infected with WT (Fig 3B), suggesting that Stx may inhibit production of IFNγ. Reduction in IFNγ-mediated STAT-1 phosphorylation has been linked to Stx activity in vitro [59]. In vivo, IFNγ is produced mainly by Th1 cells, ILC1s, NK cells and macrophages in the lamina propria. IFNγ signaling stimulates transcription of ISGs, many of which were found to be differentially expressed in animals infected with WT vs ΔΔ*stx* (Figs 2 and 3). One of those genes, *CXCL11*, is an IFNγ-inducible chemokine produced by macrophages that acts as a chemoattractant for CXCR3+ T cells; macrophage engagement of CXCR3+ T cells promotes Th1 cell development. RNA FISH analyses revealed that the MFI of the *CXCL11* signal was more intense and detected in more tissue area in samples from animals infected with ΔΔ*stx* vs WT (Fig 6A–6C). Approximately 50% of the *CXCL11* transcript signal was found in epithelial cells, with the remainder in E-cadherin negative cells in the lamina propria (Fig 6D and 6E). These observations suggest that Stx inhibits IFNγ signaling in the colonic epithelium during EHEC infection.

## Discussion

Orogastric inoculation of infant rabbits with EHEC leads to diarrheal disease and colonic pathology that closely resembles many of the intestinal aspects of human EHEC infection [21,23,46]. Here, we used this model to study how Stx modifies the host response to infection in the colon by comparing the histopathology and transcriptional profiles of the colonic mucosa from animals infected with WT EHEC or an isogenic mutant lacking Stx genes (ΔΔ*stx*). We found that Stx, a potent toxin, increases apoptosis and hemorrhage in colonic tissue and dramatically remodels the colonic epithelium’s transcriptional response to EHEC infection. Although the transcriptional responses elicited by WT and ΔΔ*stx* EHEC infection exhibited some overlap, particularly in pathways commonly associated with gram-negative infection, these isogenic strains provoked very distinct transcriptional profiles in the colonic epithelium. Nearly twice as many genes were differentially regulated in animals infected with WT/mock vs ΔΔ*stx*/mock. Moreover, differences in the epithelial response attributable to Stx were particularly prominent in immune signaling and coagulation pathways, suggesting that this toxin fundamentally modifies the intestinal innate immune response to EHEC.

The host response to EHEC infection and Stx has been difficult to study directly in humans. Examination of patient sera and urine have revealed that severe illness is associated with an increase in cytokine production, including CCL2, CCL4, CXCL1, CXCL5, CXCL8, CSF3, IL6, TNFα [60–65]. The cellular response to Stx has been characterized more extensively in tissue-cultured cells. The ‘ribotoxic stress response’ to Stx-mediated cellular damage leads to the upregulation of a variety of genes and production of proteins which modulate the immune response [19,20,66]. Purified Stx promotes the production of transcription factors (including JUN and FOS) and inflammatory cytokines (CCL2, CCL3, CCL4, CCL5, CSF2, CSF3, CXCL1, CXCL2, CXCL3, CXCL5, CXCL8, IL10, IL1αβ, IL6, TNFα) in cultured epithelial and immune cells [35,34,36,17,33,22,37–40,20]. We found that several of these genes, including *JUN*, *FOS*, *CCL4*, *CXCL8*, and *IL1A* were differentially expressed in WT vs ΔΔ*stx* infected colons; thus, Stx by itself, in the absence of additional EHEC-derived factors may account for a subset of the transcriptional changes we identified in the colonic epithelium of animals infected with the WT strain. However, recent transcriptomic studies of the response of human intestinal organoids did not detect differential expression of many of the coagulation-associated or immune signaling genes (including *F3*, *CXCL8*, and *IL23A*) that we found in infected infant rabbits [40,44]. These differences are potentially explained by organoid culture conditions, which can limit cytokine expression [67]. We detected expression of *IL23A* and *F3* in response to WT EHEC and pure Stx2 in cultured human epithelial cells, so it is unlikely the discrepancies in the infant rabbit and organoid transcriptomic studies solely reflect species-specific differences in gene expression. Overall, these discrepancies highlight the important differences in gene expression patterns observed in cultured cells and the highly complex milieu of the host intestine, and that use of purified toxin is not sufficient to capture the intricacies of the host response to a pathogen with a variety of immunomodulating signals such as LPS and T3SS effectors.

Comparisons of the epithelial transcriptional profiles induced by WT and ΔΔ*stx* EHEC in rabbit colons suggest that Stx alters the expression of important cytokines in unanticipated ways. The expression of *IL23A*, the p19 subunit of the cytokine IL23, was markedly higher in WT than ΔΔ*stx* infected colons. IL23 is thought to stimulate type 3 immune responses to extracellular pathogens, but several of our findings argue against classifying the responses we observed as type 3 immune signaling. First, IL23 is generally thought to be produced by dendritic cells [49]. Unexpectedly, nearly all the *IL23A* signal was detected in colonic epithelial cells in rabbit colons. Second, we did not detect epithelial transcripts for *IL12B*, which encodes for p40, the other subunit of the IL23 protein. Typically, the two subunits are expressed together and form the IL23 heterodimer [68], but the gene expression of these two subunits is not always temporally synchronized [69]. Third, we did not detect transcripts for *IL17A*, a cytokine induced by IL23, in the lamina propria cell fraction. Thus, at least at the time point of our transcriptional profiling, we cannot classify the Stx-driven response to EHEC as canonical type 3 immune signaling. Deciphering the functions of *IL23A* in the absence of *IL12B* may reveal an unrecognized aspect of epithelial mucosal immunity. One possibility is that p19 has an independent function apart from that of the IL23 p19-p40 heterodimer; instead, there may be alternative binding partner(s) for p19 in intestinal epithelial cells, as has been suggested recently [70].

Stx also appears to inhibit expression of *IFNG* and many ISGs such as *CXCL11*, since these cytokines and downstream factors were downregulated in WT compared to ΔΔ*stx* infected colons. Previous in vitro studies have also shown that Stx can suppress IFNγ-mediated signaling by blocking phosphorylation of STAT-1 [59] and circulating IFNγ is low in human patients infected with EHEC [61]. In contrast, A/E pathogens that lack Stx, like *Citrobacter rodentium* and EPEC, activate expression of IFNγ during infection [71–74]. IFNγ is typically expressed as part of the type 1 immune response [49], and analysis of specific immune cell populations during *Citrobacter rodentium* infection of the murine colon has revealed that this pathogen induces a robust type 1 response that is important for clearing the infection [71–73,75]. The lack of *IFNG* signaling in EHEC infected colons implies that this type of immune activation is likely not happening in the presence of Stx. Other bacterial toxins with different mechanisms of action, such as the *Clostridioides difficile* toxin and the heat-labile enterotoxin of *E*. *coli*, have also been shown to modulate the abundance and activity of various immune cell subtypes [76,77]. Our findings suggest that Stx has a similar effect on the colonic mucosa. Future work should quantify the abundance of immune cell subtypes, such as Th17, Th1, and ILCs during EHEC infection with and without Stx. However, reagents are currently not available for immune profiling in rabbits. Availability of improved reagents for cell identification will also enable refinement of cell enrichment protocols for future transcriptomic studies on purified cell populations.

Stx-mediated damage to endothelial cells, particularly in the renal microvasculature, is a well-studied hallmark of HUS. Toxin damage to endothelial cells triggers the coagulation cascade, which leads to thrombosis with fibrin deposition and hemolysis of RBCs [65]. In the colon, patient biopsies have revealed that EHEC infection can also induce microvascular thrombi and fibrin deposition [6], but few studies have investigated the patterns of gene expression which may contribute to thrombosis in the intestine. Although we were unable to detect fibrin deposition in tissue sections, we saw evidence of vascular damage in the form of hemorrhage. Hemorrhage and edema were more prominent in the WT infected vs the ΔΔ*stx* infected tissue, but the ΔΔ*stx* samples had detectable pathology. Thus, there may be both toxin-dependent and independent mechanisms that lead to increased edema and vascular damage. It may be possible to dissect the roles of other EHEC virulence factors, such as T3SS effectors, on the ability of the pathogen to cause hemorrhage using the infant rabbit model.

We also found that *F3*, the gene encoding the initiator of the coagulation cascade, is dramatically induced in colonic epithelial cells in WT but not ΔΔ*stx* infection. Similarly, *F3* transcripts were induced in tissue cultured HT29 by WT but not ΔΔ*stx* EHEC. Stx2 itself appears to be sufficient to stimulate F3 expression in epithelial cells because we found that addition of exogenous Stx2 to tissue cultured HT29 cells, a human colon cancer cell line, induced expression of *F3* transcripts to similar levels observed with WT EHEC infection. Tissue Factor is typically expressed on cells which are not in contact with blood, such as epithelial cells, and can be induced in response to inflammatory stimuli [78]. In endothelial cells, Shiga toxin can promote Tissue Factor activity through a mechanism associated with a decrease in expression of Tissue Factor Protein Inhibitor (TFPI) [54–56,58]. However, in rabbit colonic epithelial cells and in HT29 cells, *TFPI* gene expression was not altered despite a marked increase in Tissue Factor expression, suggesting an alternate mechanism of Tissue Factor induction in colonic epithelial cells. Our tissue culture experiments provide evidence that induction of *F3* in colon cells is a direct effect of Stx, but we cannot rule out the possibility that damage to endothelial cells by Stx influences the expression of this gene in vivo.

Collectively, our findings reveal that Stx powerfully shapes the host response to EHEC. Though the degree of epithelial transcriptional remodeling by Stx is striking, it is not immediately apparent whether or how this re-programing benefits the pathogen. Stx does not contribute to the ability of EHEC to colonize the infant rabbit intestine, but it does in other animal models [79–81], and it is possible Stx modulation of host gene expression enhances the pathogen’s capacity to colonize the human intestine. It is also possible that Stx-driven inflammation augments diarrhea and thereby enhances pathogen dissemination. Similarly, Stx shaping of the early innate immune response to infection likely has implications for the development of adaptive immune responses and the host’s capacity to clear the infection. Additionally, altered gene expression patterns during EHEC infection related to coagulation and inflammation are suggestive of ‘thromboinflammation,’ a mechanism in which thrombosis and inflammation synergize to contribute to disease pathology [82].

Finally, our findings illustrate the potency of combining isogenic pathogen mutants with cellular compartment-specific characterization of host transcriptional responses to infection for unravelling how individual virulence factors contribute to the cell type-specific pathogen-host dialogue in disease. For EHEC infection, understanding this dialogue and the innate immune processes contributing to the early colonic phase of disease prior to HUS could offer valuable clues for developing new therapies. Immune cell activation in the colon by Stx is hypothesized to be critical to initiate pathology in the kidneys [83]. Anti-cytokine and anti-inflammatory therapeutics have been suggested as a way to minimize immune cell activation that can lead to renal damage [84]. Future studies of the immune activation triggered by Stx damage in the colon could unlock novel targets for such therapies.

## Materials and methods

### Ethics statement

Animal experiments were conducted using protocols approved by Brigham and Women’s Hospital Committee on Animals (Institutional Animal Care and Use Committee protocol number 2016N000334 and Animal Welfare Assurance of Compliance number A4752-01) and in accordance with recommendations in the National Institute of Health’s Guide for the Care and Use of Laboratory Animals and the Animal Welfare Act of the United States Department of Agriculture.

### Bacterial strains and growth conditions

Bacterial strains were cultured in LB medium or on LB agar plates at 37°C. A gentamicin-resistant mutant of *E*. *coli* O157:H7 strain EDL933 (Δ*lacI*::*aacC1*) [85] was used in all experiments in this study and gentamicin (Gm) was used at 10 μg/mL. The ΔΔ*stx* mutant was constructed using lambda red recombineering [86] as described [87].

### Infant rabbit infection and tissue processing

Two-day old litters of mixed gender New Zealand White rabbits were co-housed with a lactating dam (Charles River). Infection inocula were prepared by diluting 100 μl of overnight culture into 100 mL of LB Gm; then, following 3 hours of growth at 37°C with shaking, 30 units of culture at OD_600_ = 1 (about 8 mL) were pelleted and resuspended in 10 mL PBS. Dilutions of the inoculum were plated to enumerate CFU. Each infant rabbit was orogastrically inoculated with 500 μl of the inoculum (~1x10^9^ CFU), using a size 4 French catheter. Following inoculation, the infant rabbits were monitored at least 2x/day for signs of illness and euthanized 2 days (36–40 hours) post infection, when the entire intestinal tract was removed.

One cm sections of the medial and distal colon were removed post necropsy and the tissue pieces were homogenized in 1 mL of sterile PBS using a minibeadbeater-16 (BioSpec Products, Inc.). Dilution series of the homogenates were plated on LB Gm plates, which were incubated overnight at 37°C, to determine CFU/g bacterial burdens in tissue sections.

### Tissue preservation and histopathology

Two cm sections of medial and distal colon were fixed in 2 mL 10% neutral-buffered formalin overnight (~16 hours) at room temperature. The next day, tissue sections were transferred to 2 mL 70% ethanol. Formalin-fixed, paraffin embedded 5 μm sections were stained with hematoxylin and eosin (H&E) by the Rodent Histopathology Core at Dana Farber Cancer Institute. Slides were blindly evaluated by a histopathologist and scored semi-quantitatively. Sections were evaluated for inflammation using the following criteria: 0, none; 1, mild infiltration of immune cells into lamina propria; 2, moderate infiltration; 3, extensive infiltration; 4, severe and extensive infiltration. Apoptosis was evaluated using the following criteria: 0, none; 1, few cells observed with fragmented nuclei; 2, many cells with fragmented nuclei; 3, significant apoptotic nuclei and penetration to crypts; 4, transmural apoptosis. Edema, congestion and hemorrhage were evaluated using the following criteria: 0, none; 1, mild vascular congestion and/or mild edema; 2, moderate congestion and/or edema; 3, congestion with hemorrhage +/ edema; 4, congestion with severe multifocal hemorrhage +/- edema. Heterophil infiltration was evaluated using the following criteria: 0, none; 1, scattered individual heterophils or small clusters in the lamina propria; 2, multifocal aggregates in mucosa with few cells in lumen; 3, multifocal aggregates in mucosa with abundant cell extrusion into lumen; 4, multifocal aggregates in mucosa with large heterophilic intraluminal rafts. Sloughing was evaluated using the following criteria: 0, none; 1, few epithelial cells sloughed from luminal surface; 2, moderate number of epithelial cells sloughed from luminal surface; 3, epithelial surface is severely disrupted; 4, extensive and severe sloughing (epithelial layer is absent). Scores were compared between infection types using a two-tailed Mann-Whitney U statistical test. The Bejmamini-Hochberg Procedure was used to control for the false discovery rate with multiple comparisons at 20%. P-values were considered significant at less than 0.05 (*), 0.01 (**), and 0.001 (***).

### Tissue preparation for RNA-sequencing

Five cm sections from between the medial and distal colon were harvested and processed immediately post necropsy for RNA sequencing from 3 rabbits inoculated with PBS (mock), WT or ΔΔ*stx* EHEC. Epithelial cell and lamina propria cell fractions were isolated from tissue using a method similar to that described previously [88]. First, fat was trimmed from the tissue, and luminal contents were gently pressed out. The tissue was then cut longitudinally and rinsed in 1 mL of wash solution (RPMI 1640, 2% Fetal Bovine Serum (FBS), 10mM HEPES, and 100 μg/mL penicillin-streptomycin). Next, the tissue was rinsed in 40 mL ice-cold Ca/Mg-free HBSS before being transferred to 10 mL of epithelial dissociation solution (HBSS, 100 μg/mL penicillin-streptomycin, 10 mM HEPES, 2% FBS, 10mM EDTA) freshly supplemented with an additional 100 μL of 0.5M EDTA. To remove dying and dead epithelial cells, the tissue was incubated in epithelial dissociation solution 37°C at 125 rpm for 5 minutes, then incubated on ice for 5 minutes, then shaken vigorously 10 times and vortexed for 2 seconds. Supernatants were discarded and the tissue piece was transferred into a new tube of 10 mL of epithelial dissociation solution freshly supplemented with an additional 100 μL of 0.5M EDTA. The solution was brought to room temperature quickly by briefly warming in a 37°C bath, then incubated for 20 minutes at 37°C at 125 rpm centrifugal rotation, then incubated on ice for 5 minutes, shaken vigorously 15 times, and vortexed vigorously for 10 seconds. The supernatant was transferred to a fresh tube and centrifuged at 300xg for five minutes. The cell pellet was resuspended in 2 mL Trizol and the solution was stored at -80°C until RNA extraction.

After epithelial cell dissociation, the remaining tissue piece was transferred to a tube containing 5 mL of enzymatic digestion solution (RPMI 1640, 2% Fetal Bovine Serum (FBS), 10mM HEPES, and 100 μg/mL penicillin-streptomycin, fresh 100 μg/mL Liberase TM, fresh 100 μg/mL DNaseI) and incubated at 37°C with centrifugal rotation at 125 rpm for 30 minutes. The digestion was quenched by adding 80 μL of 0.5M EDTA. The solution was filtered through a 40 μm cell strainer and rinsed with HBSS to a final volume of 30 mL. This tube was spun down at 400xg for 10 minutes, and the cell pellet was resuspended in 2 mL Trizol. Samples were stored at -80°C until RNA extraction.

### RNA extraction and mRNA seq library preparation and sequencing

RNA was extracted from Trizol using the Direct-Zol RNA MiniPrep Plus kit from Zymo with some modifications. First, Trizol samples were incubated at 65°C until just thawed (5–10 minutes). One mL samples were added to RNase-free microcentrifuge tubes and 200 μL of chloroform was added to each tube. The tubes were inverted 10x for mixing, and incubated at room temperature for 3 minutes. The samples were spun at 12,000xg for 15 minutes at 4°C, to separate the aqueous and organic layers. The clear aqueous phase was removed, an equal volume of 100% ethanol was added, and the sample was mixed by inversion ten times before incubating at room temperature for 5 minutes. The entire volume was transferred to a Direct-Zol spin column and spun for one minute. The samples were washed with pre-wash buffer twice, with wash buffer once, spun empty to remove residual buffer twice, and eluted with 50 μL RNase-free water. Total RNA was assessed for quality and integrity (RINe) using a High Sensitivity RNA ScreenTape (Agilent) at the HMS Biopolymers Facility.

RNA of high quality (RINe>8) was prepared for mRNA-sequencing using the KAPA mRNA HyperPrep kit (Roche). Libraries were quantified using a High Sensitivity D1000 ScreenTape (Agilent) and High Sensitivity Qubit. Libraries were sequenced on a NextSeq 550.

### Differential expression analysis

First, resources for the rabbit genome were compiled. We concatenated FASTA files for each rabbit chromosome, mitochondrial DNA, and unplaced scaffolds from OryCun2.0 (Assembly GCA_000003625.1) to create a reference genome FASTA file. The Ensembl annotation (May 2019) was used. Sequencing reads and genome resources were uploaded to the Galaxy web platform [89], and the public server usegalaxy.org was used to process and map reads. First, reads were trimmed using Trim Galore! (Galaxy Tool version 0.4.3.1) with automatic adapter sequence detection. Then, trimmed reads were mapped to the rabbit reference genome and annotation using RNA STAR (Galaxy Tool version 2.6.0b-1). featureCounts (Galaxy Tool version 1.6.4+galaxy1) was used to build a count matrix from mapped reads using the Ensembl annotation as a guide. Count matrices were exported from Galaxy and imported into R (version 3.5.3) [90]. The read counts for each gene were normalized to Transcripts Per Kilobase Million (TPM) to compare expression of marker genes across samples. TPM was calculated by first dividing the number of read counts by the length of the gene in kilobases to yield reads per kilobase (RPK). Gene RPKs were summed for each sample and this number was divided by 1,000,000 to yield a sample-specific scaling factor. The RPK value for each gene was divided by the scaling factor to yield TPM. Marker gene transcript abundance was compared using a two-tailed Mann-Whitney U non-parametric test. The Bejmamini-Hochberg Procedure was used to control for the false discovery rate with multiple comparisons at 5%. The non-normalized count matrix was also analyzed using DESeq2 (version 1.22.2) [91] to compare the abundance of transcripts between different inoculum types to identify differentially expressed genes. Parametric dispersion was used and shrinkage of effect size was performed using the package apeglm [92]. Genes with an adjusted p-value of less than 0.05 were considered to be differentially expressed. Normalized read counts were generated using a regularized log (rlog) transformation and used to perform principal component analysis (PCA) of each sample. Raw reads and count matrices have been deposited into the Gene Expression Omnibus (GEO) repository (GSE156056).

### Hierarchical clustering

For each gene, the parameter ‘rank’ was calculated by multiplying the sign of the fold-change by the log10 transformed adjusted p-value from DESeq2. Genes were ordered by rank, and hierarchical clustering of samples was performed using Euclidian sample distances using the gplot (version 3.0.1) function Heatmap.2 using r-log transformed read counts, constructing a dendrogram by column (samples). Heatmaps were scaled by row and colors assigned using ColorBrewer palette RdYlBu (version 1.1.2) [93]. Similar clustering analysis was performed for the top 50 genes by rank, and genes in the leading-edge subset from GSEA.

### Gene set enrichment

Gene set enrichment was performed using fast GSEA (fGSEA) in R (version 1.8.0) [94]. Only genes with annotation were considered. Genes were ranked with the parameter ‘rank’. The hallmark gene sets [95] and immunological signatures gene sets [51] from MSigDB [96] were used. 1000 permutations were completed, and categories with less than 10 genes and greater than 500 genes were excluded. Pathways were considered to be significantly enriched if the adjusted p-value was less than the false-discovery rate of 5%. Enrichment scores were plotted using the plotEnrichment function in R.

### Comparing differentially expressed genes in infected vs uninfected tissue

Genes with an adjusted p-value of <0.05 and log2 fold-change of >2 or <-2 from DESeq2 (WT vs mock, ΔΔ*stx* vs mock) were considered differentially expressed. These lists were compared using the online tool BioVenn [97]. The list of commonly differentially expressed genes from these comparisons was analyzed using the webtool g:Profiler to perform functional enrichment analysis (g:GOst) [98].

### Fluorescent *in situ* RNA hybridization

Fluorescent in-situ hybridization (FISH) was performed using RNAscope (ACDBio) Multiplex Fluorescent V2 Assay in 5 different rabbit colon tissue sections per inoculum type (WT, ΔΔ*stx* and mock). Manufacturer’s protocols were followed and the RNAscope HybEZ oven was used for all incubations. Freshly sectioned formalin-fixed, parrafin embedded (FFPE) tissue sections (5 μm) were processed following manufacturer’s protocol. Briefly, sections were treated with boiling target retrieval buffer for 15 minutes and digested with Protease Plus for 28 minutes. Custom RNAscope C1 probes for rabbit mRNA *IL23A*, *CXCL8*, *CXCL11*, *F3*, or *dapB* (negative control) were hybridized to the tissue. The C1 probe was detected with Opal 570 dye (Akoya Biosciences) diluted 1:1000 in Multiplex TSA buffer (ACDBio). Following completion of the RNAscope assay, sections were processed further for immunofluorescence. Sections were permeabilized with 0.1% TritonX100 for 10 minutes at room temperature, washed, and treated with 5% BSA for 1 hour to block non-specific signal. Sections were washed and stained with primary antibody to E-cadherin (1:100 anti E-cadherin mouse monoclonal antibody, BD Biosciences 610181) and/or primary antibody to O157-antigen (1:2000 anti *E*. *coli* O157:H7 goat polyclonal antibody, Abcam ab30521) overnight at 4°C. The next day, sections were washed and stained with an anti-mouse secondary antibody conjugated to Alexa 647 and/or anti-goat secondary antibody conjugated to Alexa 488 diluted 1:500 in PBS (Invitrogen A-21235). Sections were then stained with DAPI (2 μg/mL) for 5 minutes before mounting with ProLong Diamond antifade mountant (Thermo Fisher).

### Quantitative image analysis

Samples were imaged with a Nikon Ti Eclipse microscope equipped with a widefield Andor NeoZyla camera and a 20x objective. For H&E stained sections, Kohler alignment and white-balance was performed, and then RGB images were captured. For sections stained with fluorescent antibodies and FISH probes, 4x6 stitched images (10% overlap) were captured for each tissue section using multi-channel acquisition (blue, red, far-red) using 16-bit imaging. In ImageJ/FIJI [99], threshold values for each channel were determined using the sections stained with the negative control probe (*dapB*), which should have no signal in the red channel above background. To analyze mean fluorescence intensity (MFI), the threshold value for the blue channel (DAPI) was set using FIJI to create a binary mask. This mask was applied to the red channel (RNAscope probe), and a histogram of intensity values for pixels within this mask was recorded. The average value above background was recorded for each sample. To analyze the percentage of tissue expressing a transcript of interest, thresholds were applied to both the blue (DAPI) and red (RNAscope) channels to create binary masks (S11A and S11B Fig). The area of the DAPI mask was recorded. FIJI “create selection” tool was used to draw a selection around the DAPI area (S11C Fig). This selection was transferred to the RNAscope channel, and the area of RNAscope signal within this area was recorded (S11D Fig). We calculated area of tissue expressing signal by dividing area of RNAscope signal (RNAscope area within DAPI selection) by total tissue area (area of DAPI mask). To calculate the percent of the RNAscope signal derived from epithelial cells, we first applied a threshold to the far-red (E-cadherin) and red (RNAscope) channel to create a binary mask (S11A and S11B Fig). The area of the RNAscope mask was recorded. Then, the “create selection” tool in FIJI was used to draw a selection around the binary mask of the E-cadherin area (S11C Fig). The section was enlarged by 2μm to accommodate signal at the edge of the cells. This section was transferred to the binary masked RNAscope channel (S11D Fig). The area of RNAscope binary signal within the E-cadherin selection was recorded. We divided the area of RNAscope signal within E-cadherin selection by the total RNAscope signal to determine percentage of signal within epithelial cells. These three quantifications (mean fluorescent intensity, percent of tissue expressing signal, and percent of signal within epithelial cells) were compared for WT infected vs mock and ΔΔ*stx* infected vs mock for each probe using a two-tailed Mann-Whitney U statistical test. The Bejmamini-Hochberg Procedure was used to control for the false discovery rate with multiple comparisons at 20%. P-values were considered significant at less than 0.05 (*), 0.01 (**), and 0.001 (***).

### Tissue culture infection and RT-qPCR

Human colon colorectal adenocarcinoma cells (HT29, ATCC HTB-38) were purchased from ATCC and cultured in McCoy’s 5A Medium supplemented with 10% fetal bovine serum. Cells were grown at 37°C with 5% CO2. Two days before infection, 500,000 cells were seeded in 6-well plates so that infections occurred at approximately 75% confluency. One hour before infection, the media was changed to Dulbecco’s modified Eagle’s medium (DMEM) (4.5 g/L glucose). The bacterial inoculum was prepared by first growing EHEC strains statically in LB overnight at 37°C to OD600 of 0.6. Bacteria were resuspended in high-glucose DMEM to OD 0.5 and 45 μL of each inoculum was added to 5 wells of HT29 (MOI 10:1). To complement the ΔΔ*stx* mutant, 100 ng of pure Stx2 was added to 5 wells with the ΔΔ*stx* bacteria and 5 wells alone. 45 μL of DMEM was added to 5 wells as a mock infection (uninfected). The infections were carried out for a total of 6 hours, and the cells were washed once with DPBS and the media was replaced after 3 hours.

After the 6-hour infection, each well was washed twice with DPBS to remove serum-containing media. RNA was extracted using the RNeasy Plus Mini Kit (Qiagen) and cDNA was generated from 2 μg RNA using a High-Capacity cDNA Reverse Transcription kit (Thermo Fisher). Quantitative real-time PCR was performed using a Step One Plus Real-Time PCR machine using Taqman 2x master mix and Taqman probes for *GAPDH* (Hs02758991_g1), *IL23A* (Hs00372324_m1), *F3* (Hs00175225_m1) and *CXCL8* (Hs00174103_m1). Undiluted cDNA was used in the qPCR reactions. Expression levels were calculated using the delta-delta CT method normalized to *GAPDH*. Expression was normalized to the average expression of the 5 uninfected wells. Expression levels were compared using a two-tailed Student’s t-test. P-values were considered significant at less than 0.05 (*), 0.01 (**), 0.001 (***), and 0.0001 (****).

## Supporting information

S1 Fig Intestinal colonization of WT and ΔΔ*stx* EHEC are similar. CFU recovered from mid or distal rabbit colon 36 hours post inoculation with either WT or ΔΔ*stx* EHEC. Lines indicate geometric mean. n.s. (not significant) by a two-tailed Mann-Whitney U statistical test.
(TIF)

S2 Fig Immune cell infiltration is similar in animals infected with WT or ΔΔ*stx* EHEC. (A) Heterophil infiltration in colon sections from infant rabbits inoculated with PBS (mock), WT or ΔΔ*stx* EHEC 36 hours post inoculation. Scores for individual tissue sections are plotted along with the median (red line). Comparisons between groups was made using a two-tailed Mann-Whitney U test. P-values were considered significant at less than 0.05 (*) or 0.01 (**). n.s. indicates a non-significant difference. (B-D): Representative images from mock (score = 0), WT (score = 4), and ΔΔ*stx* (score = 2) infected colons. Scale bars indicate 50 μm. Orange box denotes inset displayed to the right. White arrows indicate heterophils. (E): Severity of hemorrhage/edema. (F-H): Example images from mock (score = 0), WT (score = 3), and ΔΔ*stx* (score = 3) EHEC-infected colons. Scale bars indicate 50 μm. Orange box denotes inset displayed to the right. White arrows indicate heterophils. Green arrows indicate lymphocytes.
(TIF)

S3 Fig Epithelial sloughing is similar in animals infected with WT or ΔΔ*stx* EHEC. Sloughing in colon sections from infant rabbits inoculated with PBS (mock), WT or ΔΔ*stx* EHEC 36 hours post inoculation. Scores for individual tissue sections are plotted along with the median (red line). Comparisons between groups was made using a Mann-Whitney U test. The Bejmamini-Hochberg Procedure was used to control for the false discovery rate with multiple comparisons at 20%. P-values were considered significant at less than 0.05 (*). n.s. indicates a non-significant difference.
(TIF)

S4 Fig Comparison of marker gene expression in epithelial and lamina propria cell fractions. Relative gene expression in transcripts per million for epithelial cell markers (A), stromal cell markers (B), immune cell markers (C) and enzymes in Gb3 synthesis (D). Values for individual rabbits are plotted with mean. Distributions are compared with a Mann-Whitney U test, p<0.05(*), 0.01 (**), 0.001 (***), 0.0001 (****).
(TIF)

S5 Fig Comparison of transcriptional profiles from colonic samples of infant rabbits inoculated with PBS (mock), WT or ΔΔ*stx* EHEC. (A, D, G, J) Average expression level (base mean) and log2 fold change of transcript abundance in colonic epithelial cells (A, D) or lamina propria (G, J) from rabbits inoculated with WT EHEC (A, G) or ΔΔ*stx* EHEC (D, J) compared to PBS (mock). Genes with significantly different (adjusted p-value < 0.05) transcript abundance are highlighted in red. (B, E, H, K) Heat map of rlog-transformed read counts from epithelial cells for 3 animal replicates (WT or ΔΔ*stx* EHEC infected) for all genes by rank. Hierarchical clustering performed using Euclidian sample distances. Rows are normalized by Z-score. The 4 panels correspond to the comparisons shown in the panels immediately above (A,D,G,J); W, WT EHEC, M, mock, ΔΔ, ΔΔ*stx* EHEC. (C, F, I, L)) Heat map of rlog-transformed read counts from epithelial cells for 3 animal replicates (WT or ΔΔ*stx* EHEC infected) top 25 and bottom 25 genes by rank. The 4 panels correspond to the comparisons shown in the panels immediately above (B, E, H, K); W, WT EHEC, M, mock, ΔΔ, ΔΔ*stx* EHEC.
(TIF)

S6 Fig WT and ΔΔ*stx* EHEC colonic colonization both stimulate host transcriptional responses commonly associated with infection. A,C) Venn diagrams of differentially expressed genes in wt vs mock infection and ΔΔ*stx* vs. mock infection from colonic epithelial cells (A) or lamina propria cells (C). B,D) Transcriptional changes elicited by both strains map to many pathways associated with infection by GO Molecular Function analysis in colonic epithelial cells (B) or lamina propria cells (D).
(TIF)

S7 Fig Infection with WT and ΔΔ*stx* led to distinct transcriptomic signatures. (A) Heat map of rlog-transformed read counts from epithelial cells for 3 animal replicates (WT or ΔΔ*stx* EHEC infected) for all genes by rank. Hierarchical clustering performed using Euclidian sample distances. Rows are normalized by Z-score. (B) Heat map of rlog-transformed read counts from lamina propria cells for 3 animal replicates (WT or ΔΔ*stx* EHEC infected) for all genes by rank. Hierarchical clustering performed using Euclidian sample distances. Rows are normalized by Z-score.
(TIF)

S8 Fig RNAscope signal is not limited to regions of bacterial attachment. Immunofluorescence micrographs of colon sections from rabbits inoculated with WT EHEC (A-C) or ΔΔ*stx* EHEC (D) stained with an RNAscope probe (red) for rabbit *F3* (A), *IL23A* (B), *CXCL8* (C), or *CXCL11* (D), DAPI (blue), an anti-O157 antibody (green), and an anti-E-cadherin antibody (white). Scale bar is 50 μM.
(TIF)

S9 Fig Pure Stx2 can induce *F3* and *IL23A* gene expression in HT29 cells in vitro. Normalized expression of *F3* (A) and *IL23A* (B) in HT29 cells infected with WT EHEC, ΔΔ*stx* EHEC, ΔΔ*stx* EHEC plus 100 ng of pure Stx2, 100 ng of pure Stx2 alone. Expression levels compared with a Students two-tailed t-test, p<0.0001 (****).
(TIF)

S10 Fig Expression of *CXCL8* in epithelial cells is much greater in animals infected with WT vs ΔΔ*stx* EHEC. (A) Micrographs of colon sections from rabbits inoculated with WT EHEC, ΔΔ*stx* EHEC, or PBS (mock) stained with a probe to rabbit *CXCL8* mRNA (red) and DAPI (blue). Scale bar is 500 μM. (B) Percentage of tissue section with *CXCL8* signal from individual colons. Distributions compared using Mann-Whitney U test, p<0.01 (**), n.s. indicates not significant. (C) Mean fluorescent intensity (MFI) from individual colons plotted with mean. Distributions compared using Mann-Whitney U test, p<0.01 (**), n.s. indicates not significant. (D) Percent *CXCL8* signal within E-cadherin positive cells. Distributions compared using the Mann-Whitney U test, p<0.05 (*). (E) Sections stained with a probe to rabbit *CXCL8* mRNA (red), DAPI (blue), and anti-E-cadherin antibody (white). Scale bar is 500 μM. Example immune cell (IC) and epithelial cell (EC) is indicated. (F) Normalized expression of *CXCL8* in HT29 cells infected with WT EHEC, ΔΔ*stx* EHEC, or PBS. Expression levels compared with a Students two-tailed t-test, p<0.001 (***), n.s. indicates not significant.
(TIF)

S11 Fig Method to assign RNA FISH signal to cellular compartments. (A) Original images collected in DAPI, E-cadherin, and RNAscope channels (B). Binary masks created using the FIJI “threshold” tool. (C) FIJI “create selection” tool was used to draw a region around binary mask. (D) Selection was transferred to RNAscope channel to determine portion of signal within region of interest. See methods for more detail.
(TIF)

S1 Table Results of DESeq2 comparing wt vs mock infected rabbit colonic epithelial cells. (XLSX)

S2 Table Results of DESeq2 comparing ΔΔstx vs mock infected rabbit colonic epithelial cells. (XLSX)

S3 Table Results of DESeq2 comparing wt vs mock infected rabbit lamina propria cells. (XLSX)

S4 Table Results of DESeq2 comparing ΔΔstx vs mock infected rabbit lamina propria cells. (XLSX)

S5 Table Results of DESeq2 and fGSEA comparing wt vs ΔΔstx infected rabbit colonic epithelial cells. (XLSX)

S6 Table Results of DESeq2 and fGSEA comparing wt vs ΔΔstx infected rabbit lamina propria cells. (XLSX)
