# Supplementary figures and images for: Shiga toxin remodels the intestinal epithelial transcriptional response to Enterohemorrhagic Escherichia coli
Source: PLoS Pathog. 2021 Feb 2;17(2):e1009290. doi: 10.1371/journal.ppat.1009290 (PMC7880444; doi:10.1371/journal.ppat.1009290)

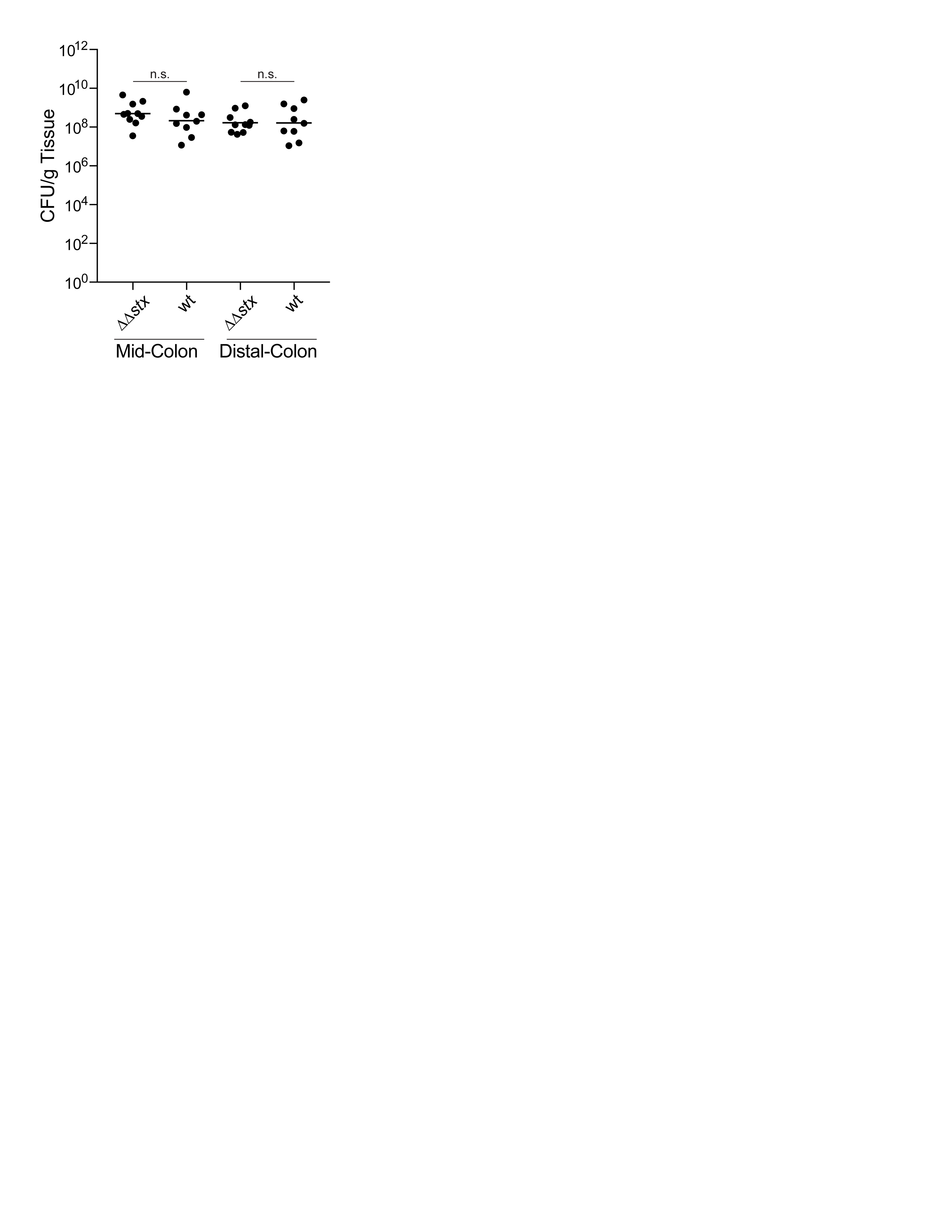

Supplement: S1 Fig — CFU recovered from mid or distal rabbit colon 36 hours post inoculation with either WT or ΔΔstx EHEC. Lines indicate geometric mean. n.s. (not significant) by a two-tailed Mann-Whitney U statistical test. (TIF) [file ppat.1009290.s001.tif]

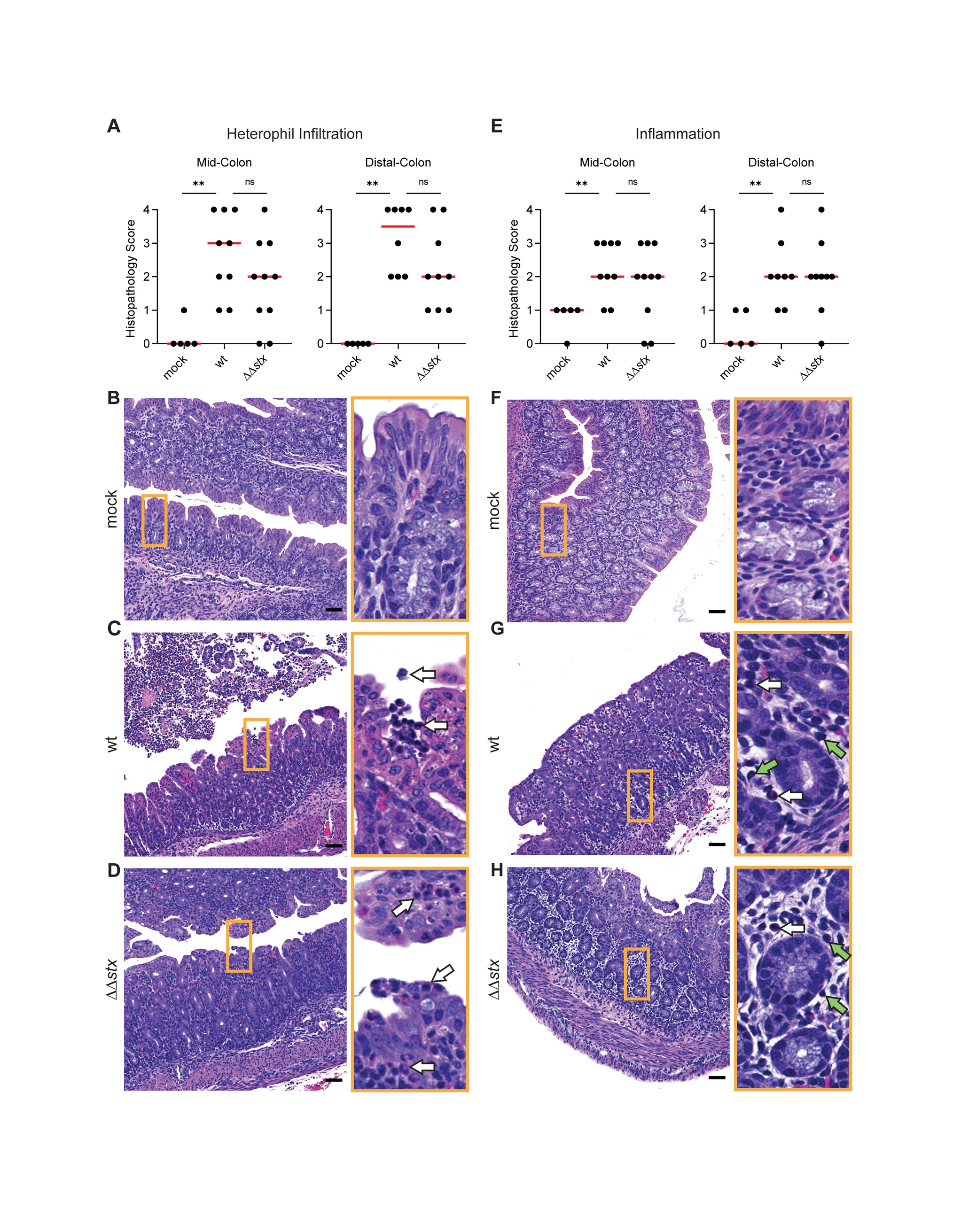

Supplement: S2 Fig — (A) Heterophil infiltration in colon sections from infant rabbits inoculated with PBS (mock), WT or ΔΔstx EHEC 36 hours post inoculation. Scores for individual tissue sections are plotted along with the median (red line). Comparisons between groups was made using a two-tailed Mann-Whitney U test. P-values were considered significant at less than 0.05 (*) or 0.01 (**). n.s. indicates a non-significant difference. (B-D): Representative images from mock (score = 0), WT (score = 4), and ΔΔstx (score = 2) infected colons. Scale bars indicate 50 μm. Orange box denotes inset displayed to the right. White arrows indicate heterophils. (E): Severity of hemorrhage/edema. (F-H): Example images from mock (score = 0), WT (score = 3), and ΔΔstx (score = 3) EHEC-infected colons. Scale bars indicate 50 μm. Orange box denotes inset displayed to the right. White arrows indicate heterophils. Green arrows indicate lymphocytes. (TIF) [file ppat.1009290.s002.tif]

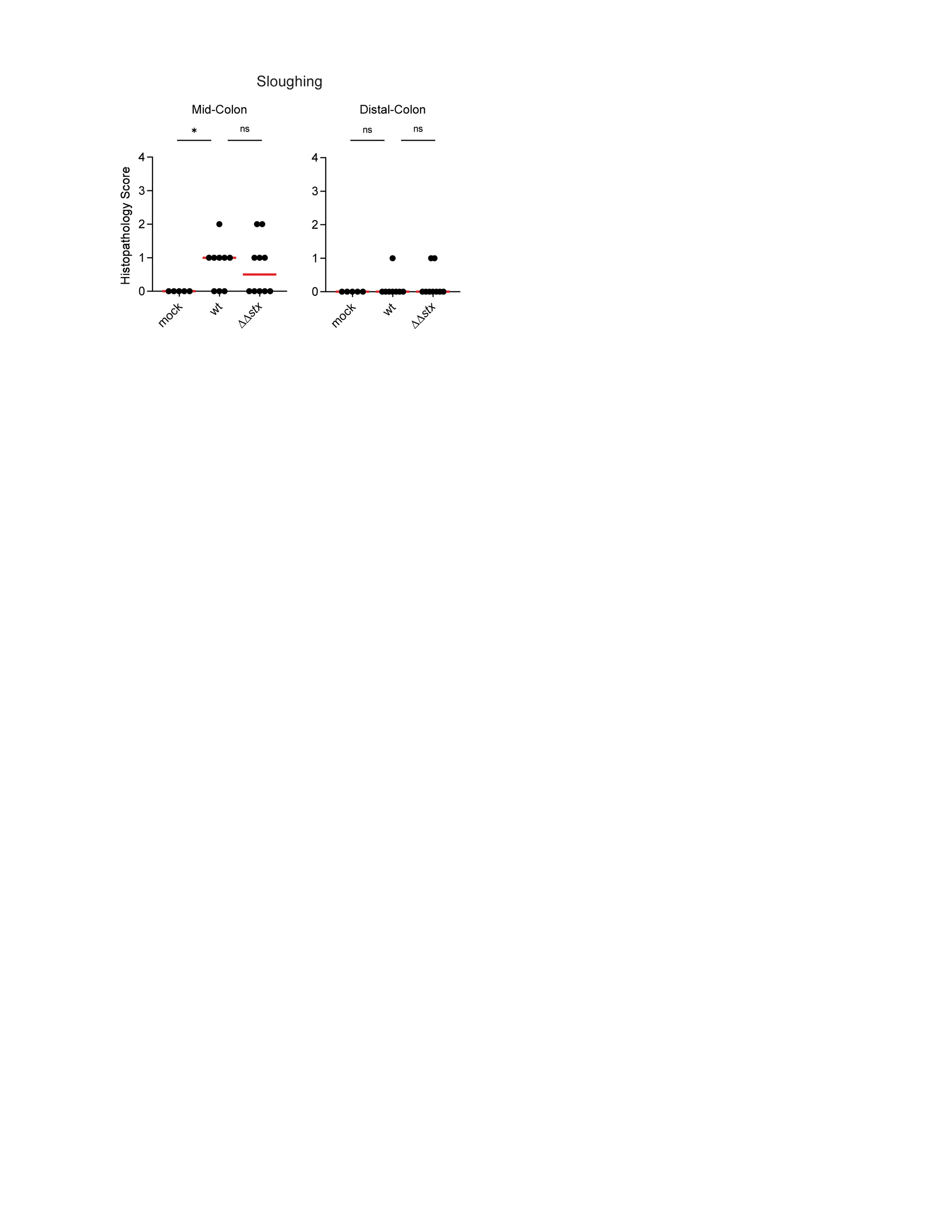

Supplement: S3 Fig — Sloughing in colon sections from infant rabbits inoculated with PBS (mock), WT or ΔΔstx EHEC 36 hours post inoculation. Scores for individual tissue sections are plotted along with the median (red line). Comparisons between groups was made using a Mann-Whitney U test. The Bejmamini-Hochberg Procedure was used to control for the false discovery rate with multiple comparisons at 20%. P-values were considered significant at less than 0.05 (*). n.s. indicates a non-significant difference. (TIF) [file ppat.1009290.s003.tif]

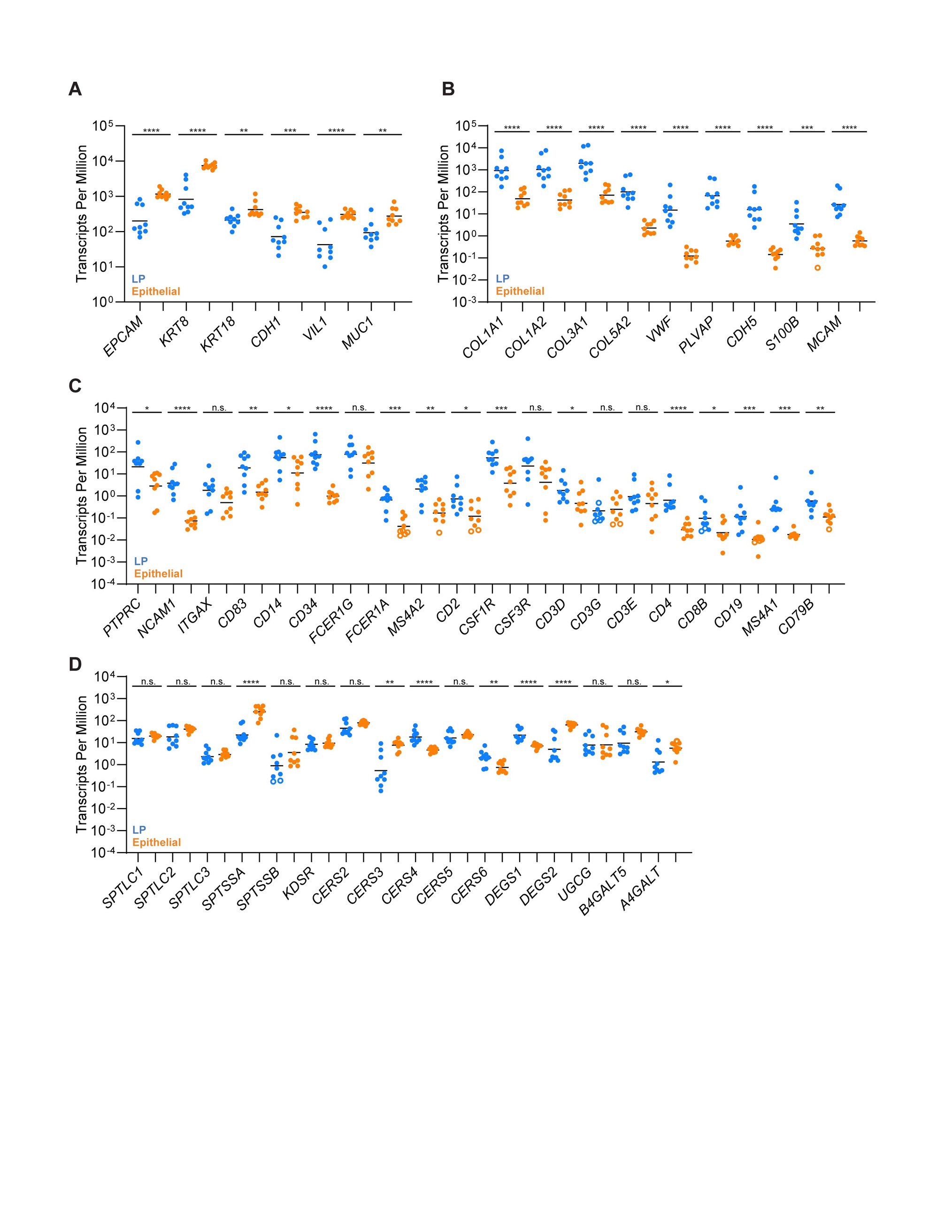

Supplement: S4 Fig — Relative gene expression in transcripts per million for epithelial cell markers (A), stromal cell markers (B), immune cell markers (C) and enzymes in Gb3 synthesis (D). Values for individual rabbits are plotted with mean. Distributions are compared with a Mann-Whitney U test, p<0.05(*), 0.01 (**), 0.001 (***), 0.0001 (****). (TIF) [file ppat.1009290.s004.tif]

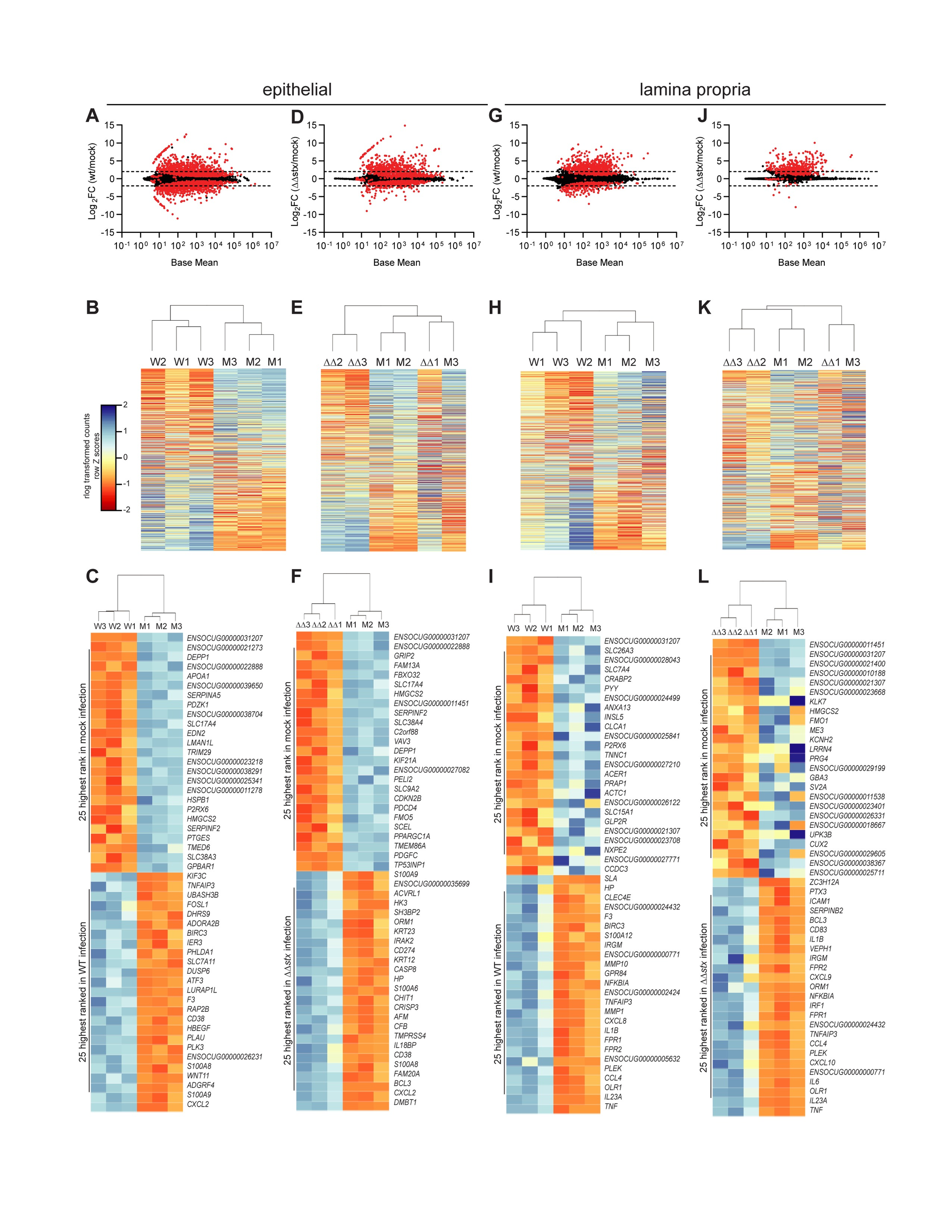

Supplement: S5 Fig — (A, D, G, J) Average expression level (base mean) and log2 fold change of transcript abundance in colonic epithelial cells (A, D) or lamina propria (G, J) from rabbits inoculated with WT EHEC (A, G) or ΔΔstx EHEC (D, J) compared to PBS (mock). Genes with significantly different (adjusted p-value < 0.05) transcript abundance are highlighted in red. (B, E, H, K) Heat map of rlog-transformed read counts from epithelial cells for 3 animal replicates (WT or ΔΔstx EHEC infected) for all genes by rank. Hierarchical clustering performed using Euclidian sample distances. Rows are normalized by Z-score. The 4 panels correspond to the comparisons shown in the panels immediately above (A,D,G,J); W, WT EHEC, M, mock, ΔΔ, ΔΔstx EHEC. (C, F, I, L)) Heat map of rlog-transformed read counts from epithelial cells for 3 animal replicates (WT or ΔΔstx EHEC infected) top 25 and bottom 25 genes by rank. The 4 panels correspond to the comparisons shown in the panels immediately above (B, E, H, K); W, WT EHEC, M, mock, ΔΔ, ΔΔstx EHEC. (TIF) [file ppat.1009290.s005.tif]

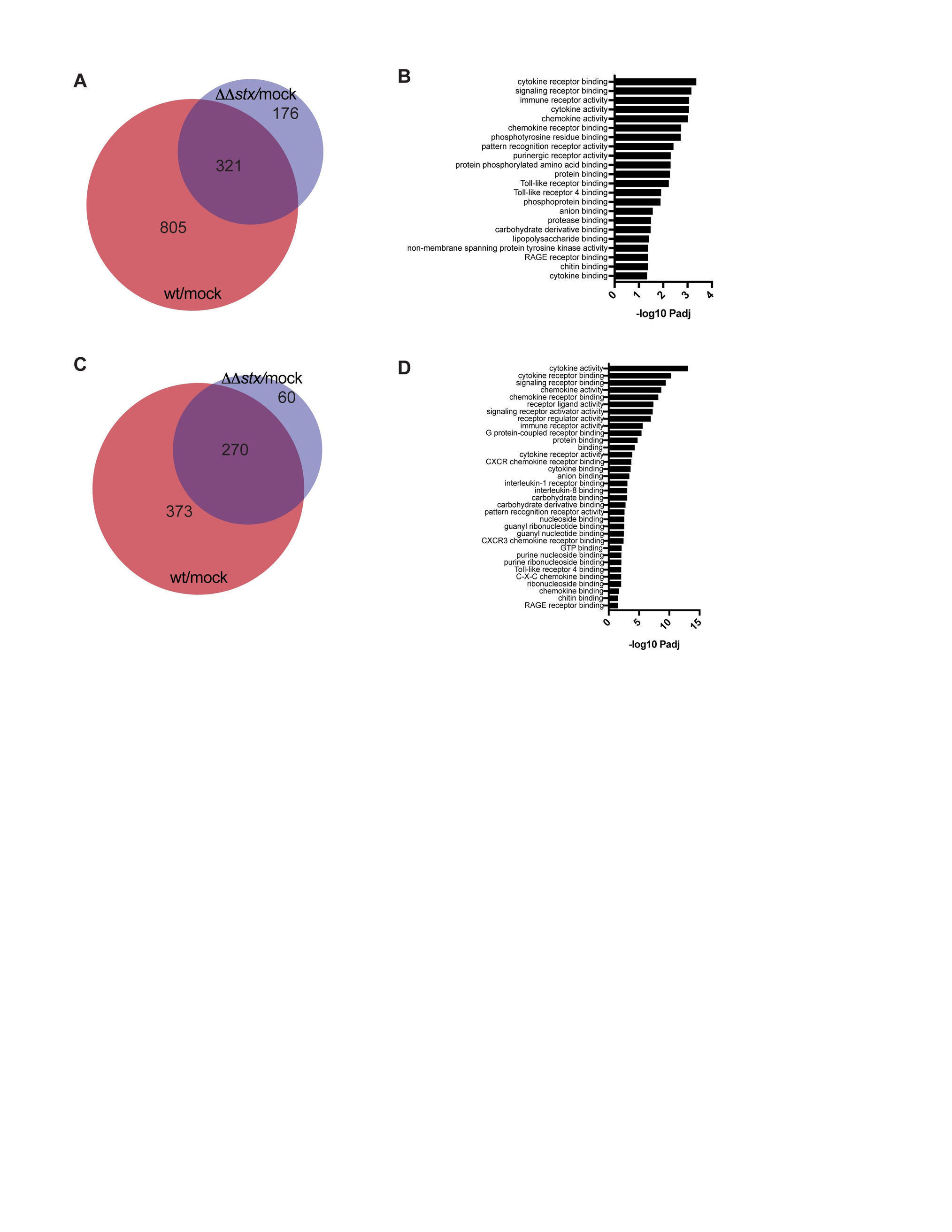

Supplement: S6 Fig — A,C) Venn diagrams of differentially expressed genes in wt vs mock infection and ΔΔstx vs. mock infection from colonic epithelial cells (A) or lamina propria cells (C). B,D) Transcriptional changes elicited by both strains map to many pathways associated with infection by GO Molecular Function analysis in colonic epithelial cells (B) or lamina propria cells (D). (TIF) [file ppat.1009290.s006.tif]

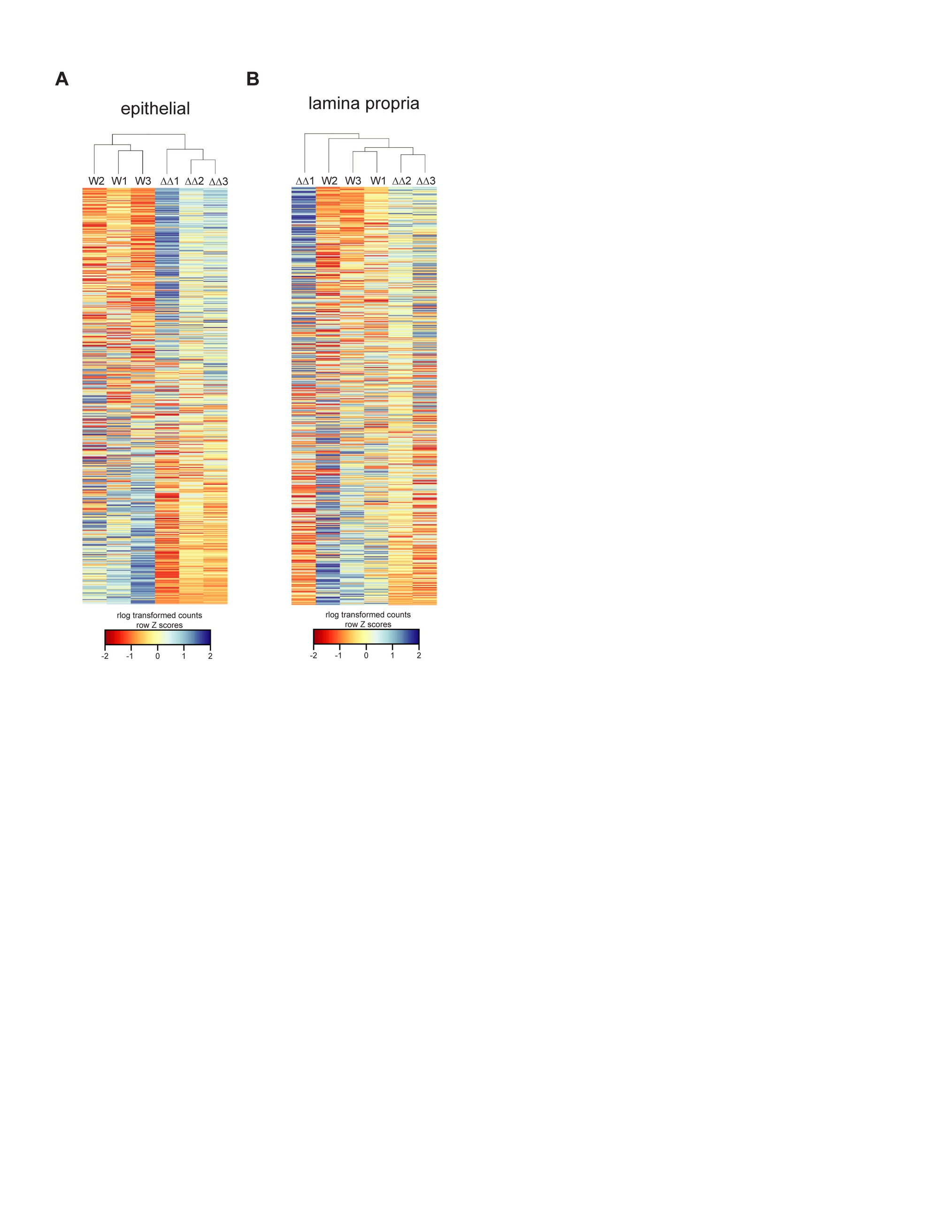

Supplement: S7 Fig — (A) Heat map of rlog-transformed read counts from epithelial cells for 3 animal replicates (WT or ΔΔstx EHEC infected) for all genes by rank. Hierarchical clustering performed using Euclidian sample distances. Rows are normalized by Z-score. (B) Heat map of rlog-transformed read counts from lamina propria cells for 3 animal replicates (WT or ΔΔstx EHEC infected) for all genes by rank. Hierarchical clustering performed using Euclidian sample distances. Rows are normalized by Z-score. (TIF) [file ppat.1009290.s007.tif]

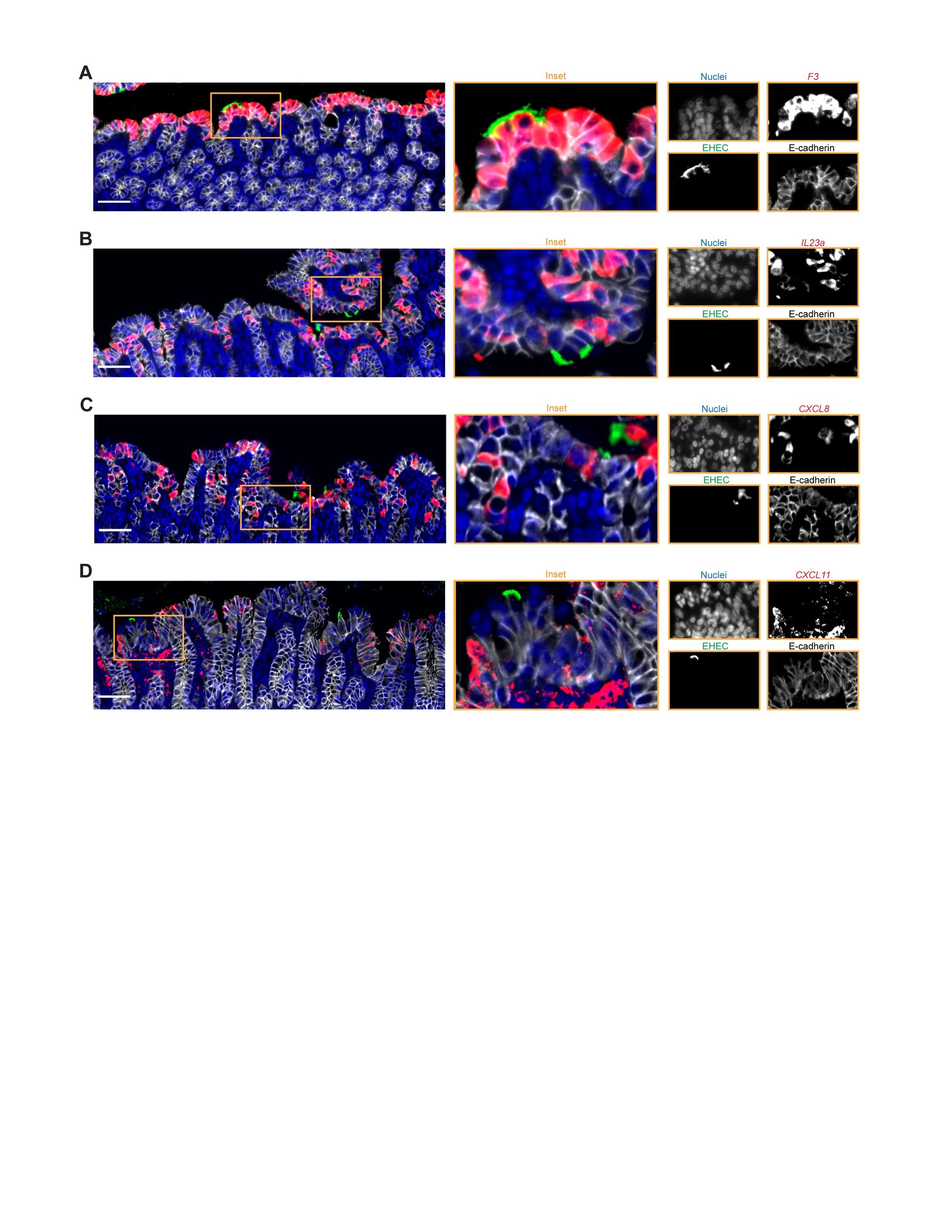

Supplement: S8 Fig — Immunofluorescence micrographs of colon sections from rabbits inoculated with WT EHEC (A-C) or ΔΔstx EHEC (D) stained with an RNAscope probe (red) for rabbit F3 (A), IL23A (B), CXCL8 (C), or CXCL11 (D), DAPI (blue), an anti-O157 antibody (green), and an anti-E-cadherin antibody (white). Scale bar is 50 μM. (TIF) [file ppat.1009290.s008.tif]

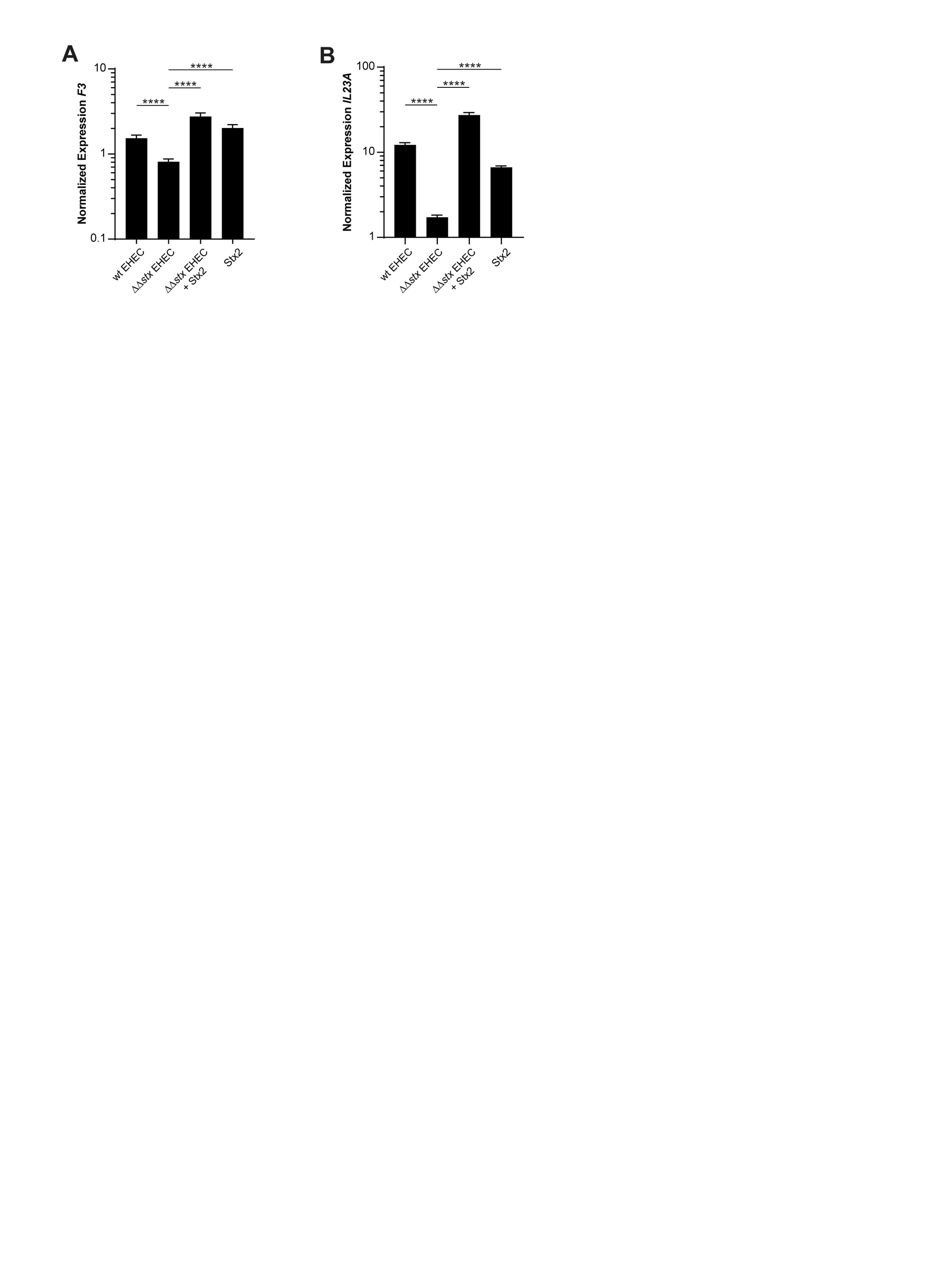

Supplement: S9 Fig — Normalized expression of F3 (A) and IL23A (B) in HT29 cells infected with WT EHEC, ΔΔstx EHEC, ΔΔstx EHEC plus 100 ng of pure Stx2, 100 ng of pure Stx2 alone. Expression levels compared with a Students two-tailed t-test, p<0.0001 (****). (TIF) [file ppat.1009290.s009.tif]

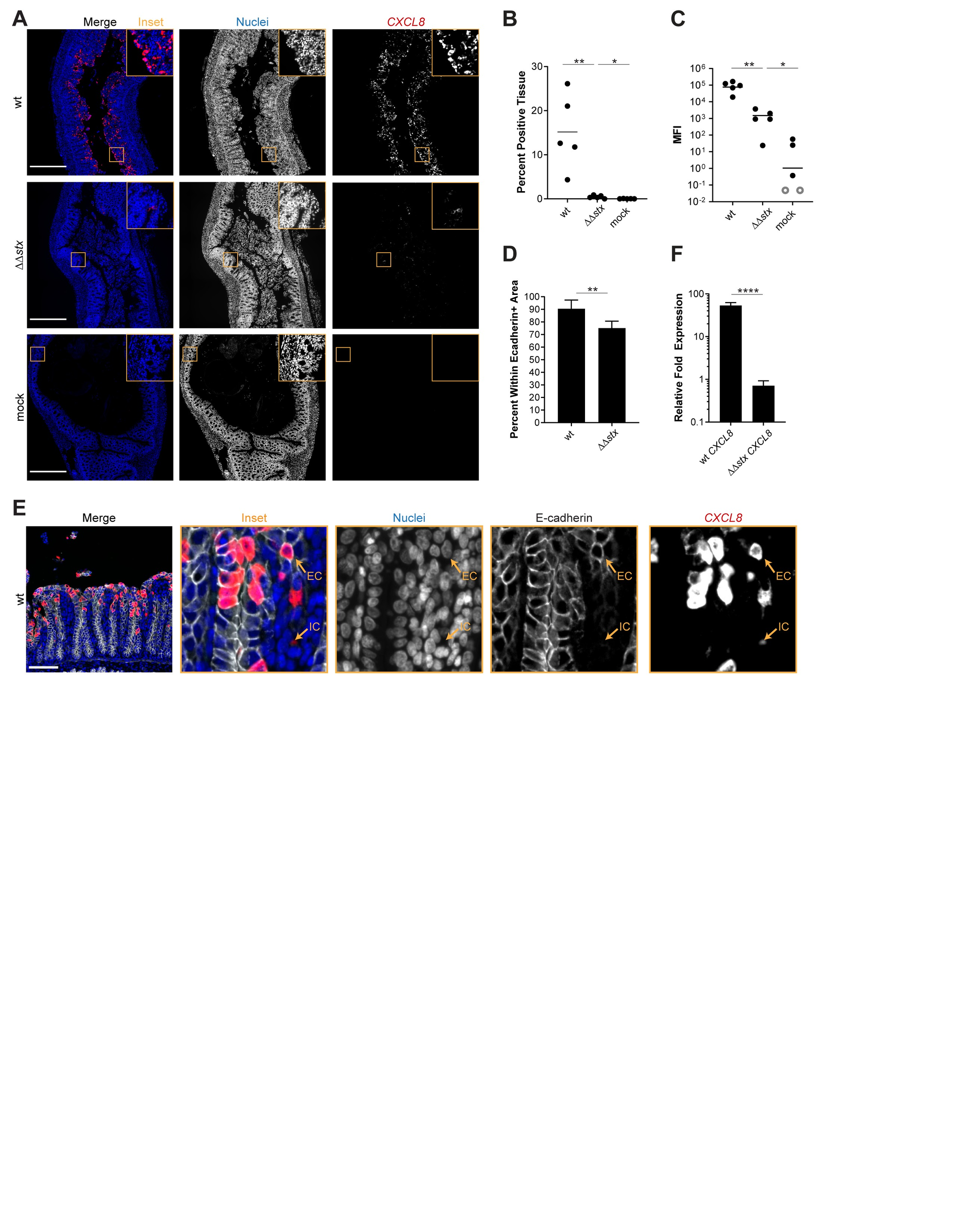

Supplement: S10 Fig — (A) Micrographs of colon sections from rabbits inoculated with WT EHEC, ΔΔstx EHEC, or PBS (mock) stained with a probe to rabbit CXCL8 mRNA (red) and DAPI (blue). Scale bar is 500 μM. (B) Percentage of tissue section with CXCL8 signal from individual colons. Distributions compared using Mann-Whitney U test, p<0.01 (**), n.s. indicates not significant. (C) Mean fluorescent intensity (MFI) from individual colons plotted with mean. Distributions compared using Mann-Whitney U test, p<0.01 (**), n.s. indicates not significant. (D) Percent CXCL8 signal within E-cadherin positive cells. Distributions compared using the Mann-Whitney U test, p<0.05 (*). (E) Sections stained with a probe to rabbit CXCL8 mRNA (red), DAPI (blue), and anti-E-cadherin antibody (white). Scale bar is 500 μM. Example immune cell (IC) and epithelial cell (EC) is indicated. (F) Normalized expression of CXCL8 in HT29 cells infected with WT EHEC, ΔΔstx EHEC, or PBS. Expression levels compared with a Students two-tailed t-test, p<0.001 (***), n.s. indicates not significant. (TIF) [file ppat.1009290.s010.tif]

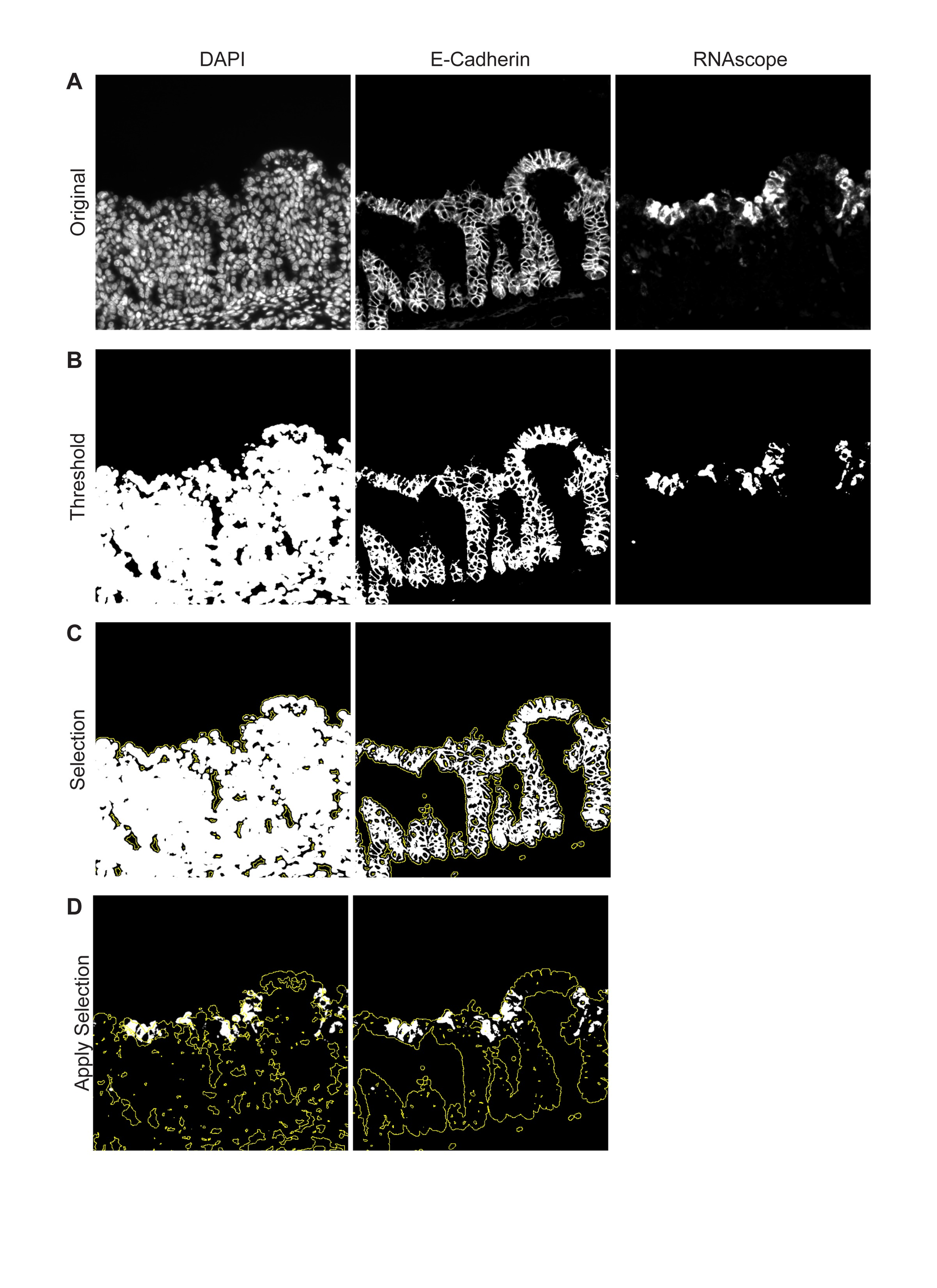

Supplement: S11 Fig — (A) Original images collected in DAPI, E-cadherin, and RNAscope channels (B). Binary masks created using the FIJI “threshold” tool. (C) FIJI “create selection” tool was used to draw a region around binary mask. (D) Selection was transferred to RNAscope channel to determine portion of signal within region of interest. See methods for more detail. (TIF) [file ppat.1009290.s011.tif]
